# Sunlight-driven dissolution is a major fate of oil at sea

**DOI:** 10.1126/sciadv.abl7605

**Published:** 2022-02-16

**Authors:** Danielle Haas Freeman, Collin P. Ward

**Affiliations:** 1 Department of Marine Chemistry and Geochemistry, Woods Hole Oceanographic Institution, Woods Hole, MA 02543, USA.; 2 Department of Civil and Environmental Engineering, Massachusetts Institute of Technology, Cambridge, MA 02139, USA.

## Abstract

Oxygenation reactions initiated by sunlight can transform insoluble components of crude oil at sea into water-soluble products, a process called photo-dissolution. First reported a half century ago, photo-dissolution has never been included in spill models because key parameters required for rate modeling were unknown, including the wavelength and photon dose dependence. Here, we experimentally quantified photo-dissolution as a function of wavelength and photon dose, making possible a sensitivity analysis of environmental variables in hypothetical spill scenarios and a mass balance assessment for the 2010 *Deepwater Horizon* (DwH) spill. The sensitivity analysis revealed that rates were most sensitive to oil slick thickness, season/latitude, and wavelength and less sensitive to photon dose. We estimate that 3 to 17% (best estimate 8%) of DwH surface oil was subject to photo-dissolution, comparable in magnitude to other widely recognized fate processes. Our findings invite a critical reevaluation of surface oil budgets for both DwH and future spills at sea.

## Teaser

Sunlight-driven dissolution is quantitatively comparable with other oil fate processes and should be included in spill models.

## INTRODUCTION

In the aftermath of an oil spill at sea, sunlight exposure can initiate a suite of chemical reactions in floating slicks that transform the oil into new compounds. These transformations include (i) photo-oxidation, which results in the incorporation of oxygen into the carbon backbone of oil hydrocarbons (*1*, *2*); (ii) fragmentation, which results in the decrease of molecular weight via chain scission reactions; and (iii) polymerization, which results in an increase in molecular weight via cross-linking reactions (*3*, *4*). These photochemical processes are a major fate pathway for surface oil. For example, during the 2010 *Deepwater Horizon* (DwH) spill in the Gulf of Mexico (GoM), about half of the residual surface oil was transformed by sunlight into new partially oxidized compounds in less than a week, as measured by oxygen incorporation (*5*). Photochemistry matters for oil spill modeling and response efforts because sunlight-generated species have distinct physical and chemical properties from crude oil (*1*, *2*), thus affecting oil transport, environmental partitioning, and the effectiveness of response tools such as chemical dispersants (*6*). Furthermore, photochemistry may have opposing effects on different pools of oil compounds depending on the relative importance of different reaction pathways (for example, oxidation, fragmentation, and polymerization), resulting in observations of both persistent, presumably high–molecular weight polymerized species [for example, tar balls on beaches (*5*, *7*–*9*)] and lower–molecular weight volatile and water-soluble species (*1*, *3*, *10*). Because oxygenation is a ubiquitous feature of both the high– and low–molecular weight materials produced by sunlight (*5*, *10*), “photo-oxidation” is commonly used to refer to the suite of photochemical processes influencing oil and will be used throughout this article.

Among the many oil properties affected by photo-oxidation is water solubility. Laboratory studies over the past 50 years have shown that light exposure can generate water-soluble products from crude oil components that were originally insoluble, a process called photo-dissolution (one of many possible photo-oxidation pathways, some of which also produce water-insoluble products) (*11*–*13*). In a spill scenario, the water-soluble photo-products partition into seawater, joining the marine dissolved organic carbon (DOC) pool. Photo-dissolution therefore constitutes a removal process for oil slicks, potentially affecting the overall mass balance of surface oil, as well as serving as a source of new oil-derived compounds to the water column with unknown effects on marine ecosystems (*10*). Thus, photo-dissolution may have a major impact on oil fate and effects, but it is not currently included in any oil fate models, nor has its impact been evaluated for any past spills (*14*–*18*). The absence of data on this process likely contributed to the conclusion of the 2003 National Academy of Sciences report that chemical transformations of oil by sunlight are “unimportant from a mass balance consideration” (*19*). Given the wide uncertainty ranges in the mass balance of even a well-studied spill, such as DwH, there is room for an unaccounted-for process (e.g., photo-dissolution) to explain gaps in a mass balance and refine estimates for other fate processes (*20*–*22*).

A quantitative evaluation of the amount of oil transformed by photo-dissolution in any given spill scenario requires data on the kinetics of this process. Rates of photo-dissolution are a function of two variables, the apparent quantum yield (AQY; mol DOC per mol photons absorbed), which is the efficiency of the reaction, and the rate of light absorption by the oil. While the absorption properties of oils are relatively well understood (*23*), AQYs for oil photo-dissolution have not been experimentally determined, preventing an evaluation of the importance of this process during a spill at sea.

Rate calculations are complicated by the fact that both AQYs and light absorption rates are sensitive to multiple variables (fig. S1). AQYs may vary as a function of oxygen availability, temperature, oil type, the wavelength of incident irradiance, and the amount of previous light exposure (i.e., the “photon dose”). Photon dose, a proxy for sunlight exposure time on the sea surface that measures the cumulative amount of light absorbed by the oil (mol photons), should be distinguished from irradiance, which measures the rate of light delivery to the sea surface (mol photons m^−2^ day^−1^), and does not account for the surface area, thickness, or absorption properties of the oil, or of the exposure time. AQYs are expected to decrease as a function of wavelength from the ultraviolet (UV) to the visible region by analogy with DOC (*24*–*26*), but neither the shape of this relationship nor the relevant wavelength range has been experimentally determined for oil photo-dissolution. Much previous work on oil photo-oxidation simply assumes an exponentially decreasing relationship between AQY and wavelength (*5*) or assumes that only UV light is relevant for photo-oxidation and photo-toxicity (*27*, *28*). Recent work showing that visible light is a major contributor to oil photo-oxidation warrants a reevaluation of this assumption when measuring AQYs (*5*). Less information is available on the relationship between AQYs and photon dose: If oil behaves analogously to DOC, we might expect AQYs to decrease as a function of increasing light exposure (*29*, *30*).

Application of AQYs determined in the laboratory to a spill scenario is further complicated by variation in light absorption rates. Like AQYs, light absorption rates also vary with wavelength and oil type. Moreover, rates of light absorption are influenced by oil slick thickness and environmental variables that control solar irradiance, such as the time of year (seasonality), latitude, and cloudiness (sky conditions). Despite knowledge of all these variables that influence oil photo-oxidation rates, no study has conducted a sensitivity analysis to determine which variables are most important in controlling oil photo-oxidation at sea.

**Fig. 1.**
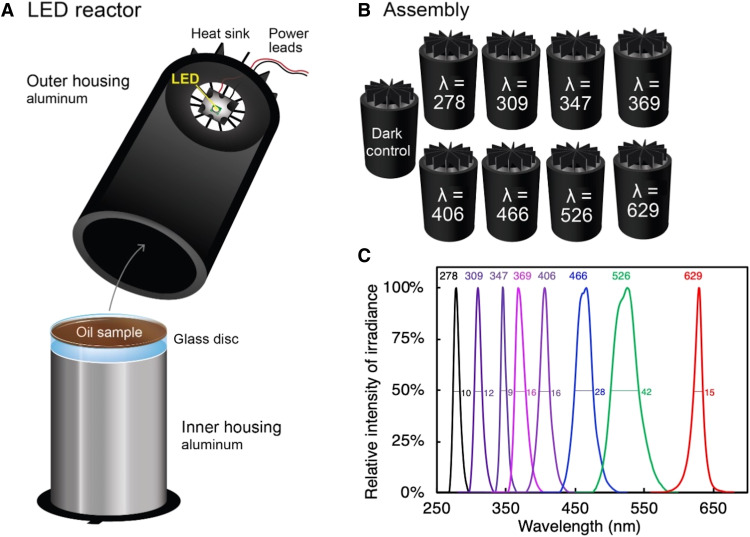
LED-based reactor system used for laboratory irradiations of oil. (**A**) Schematic of the LED reactor. Each reactor is composed of two housings, the inner and outer. The inner housing hosts an aluminum platform of varying heights with a smooth glass disk on which a uniform 200-μm-thick oil film sits. The oil film sits directly under the LED chip. The outer housing hosts the LED chip, which is mounted on a printed circuit board and a heat sink. The power leads go to a power supply (not shown). When in use, the outer housing sits snugly over the inner housing. (**B**) Reactor assembly used to irradiate oil in this study, with LEDs at wavelengths (nm) from the UV-C region (278 nm) through red light in the visible region (629 nm). (**C**) Relative spectral photon irradiance for the eight LED chips used in this study. The peak waveband and full width at half maximum of each peak (nm) are listed.

In this study, we evaluated the importance of photo-dissolution as a fate process for a single well-studied crude oil: Macondo oil (a light, sweet, Louisiana crude), which spilled during DwH (*31*). Our specific goals were to (i) experimentally quantify AQYs for photo-dissolution as a function of both wavelength and photon dose, (ii) conduct a sensitivity analysis to determine which environmental variables control photo-dissolution rates in hypothetical spill scenarios at sea, and (iii) quantify the impact of photo-dissolution on the fate of DwH surface oil. Using a custom-built light-emitting diode (LED) reactor system (Fig. 1) (*24*), we found that oil photo-dissolution AQYs decrease exponentially with increasing wavelength, reactivity extends deep into the visible light region, and AQYs decrease with increasing photon dose. The sensitivity analysis revealed that rates were most sensitive to oil slick thickness, season/latitude, and wavelength and less sensitive to photon dose. Last, kinetic rate modeling revealed that oil photo-dissolution was a major fate term in the mass balance of DwH surface oil, comparable in magnitude to other widely recognized fates such as evaporation and stranding on coastlines. Collectively, our findings call for a reevaluation of the mass balance for the DwH oil spill and provide critical empirical data that allow us to move beyond DwH and predict the importance of oil photo-dissolution for future spills at sea.

## RESULTS

### Photo-dissolution AQYs as a function of wavelength and photon dose

Photo-dissolution AQYs were experimentally determined as a function of wavelength and photon dose by irradiating uniform, 200-μm-thick oil films under narrow (~20 nm) wavebands of UV and visible light provided by custom-built LED-based reactors that allowed for easy control over irradiant output (Fig. 1) (*24*). Oil films were irradiated at a total of eight wavebands from 278 to 629 nm and four photon doses corresponding to environmental sunlight exposure times on the scale of days (for visible light) to years (for short-wavelength UV). The irradiated oil was then equilibrated with seawater. Water-soluble photo-products were operationally defined and quantified as DOC that passed through a 0.22-μm filter. AQYs were calculated for each wavelength and dose as the number of moles of DOC produced per the number of moles of light absorbed by the oil sample. The experimental results were subsequently used to conduct a sensitivity analysis (see the next section) and an assessment of the extent to which photo-dissolution may have affected the mass balance of DwH surface oil (see the “How much DwH surface oil was converted to DOC by photo-dissolution?” section).

**Fig. 2.**
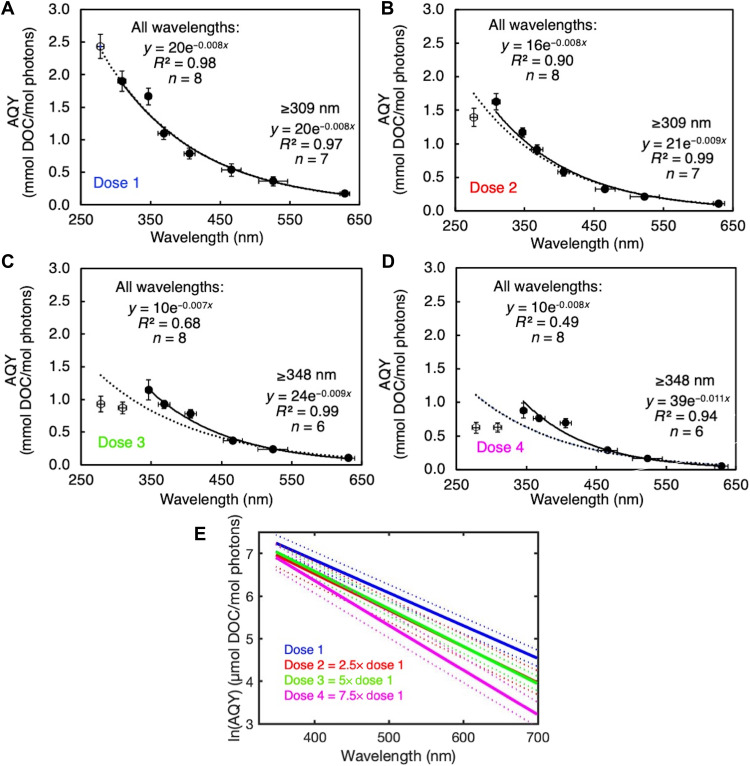
AQY spectra for the photo-dissolution of oil measured at four target photon doses. (**A**) Dose 1 = 0.011 mol photons, (**B**) dose 2 = 0.027 mol photons, (**C**) dose 3 = 0.053 mol photons, and (**D**) dose 4 = 0.080 mol photons. The experimentally determined AQY values were fit to an exponential model. For photon doses 2 to 4, one or both of the experimentally determined AQYs for 278 and 309 nm fell below the expected exponential decay model of the data. Thus, exponential regressions are shown for all data (open and filled circles; dotted regression line), as well as for wavelengths ≥309 nm or ≥348 nm (filled circles only; solid regression line). Both *R*^2^ values and *n* values for each regression are shown in each subfigure. Vertical error bars are propagated uncertainty in AQY from the SEM amount of DOC produced (*n* = 3) and the SEM moles of photons absorbed (*n* = 5). Horizontal error bars show the average full width at half maximum of the spectral output of each LED (*n* = 5). In (**E**), natural log-transformed AQY spectra are shown for all four photon doses (≥348 nm). Thick lines show the regression line, while dotted lines show the upper and lower bounds of the 95% confidence intervals for the regressions.

Under photon dose 1 conditions, AQYs for oil photo-dissolution decreased exponentially as a function of wavelength from 278 to 629 nm (Fig. 2). An exponential decay equation provided a better regression fit (*R*^2^ = 0.98, *n* = 8) than a linear regression (*R*^2^ = 0.84, *n* = 8; regression line not shown). All wavebands of light tested resulted in nonzero AQYs, indicating that all wavebands of light tested in the UV and visible range are relevant for oil photo-oxidation at sea.

Oil photo-dissolution AQYs generally decreased with increasing photon dose, but the impact was not equal across all wavelengths (Fig. 2). From doses 1 to 4, AQYs decreased substantially at all wavebands. However, higher photon doses caused a disproportionate decrease in AQYs at 278 and 309 nm relative to other wavelengths. For example, the largest decrease occurred at 278 nm (74%), whereas the smallest decrease occurred at 406 nm (12%; Fig. 2, A to D). This disproportionate effect of photon dose muddled the monotonically decreasing relationship between AQY and wavelength, showing progressively worse exponential decay regression fits when all wavelengths were included (*R*^2^ = 0.49, *n* = 8 for dose 4; Fig. 2, B to D). However, exponential regressions of only the longer wavelength data points (≥309 nm for dose 2 and ≥348 nm for doses 3 and 4) resulted in strong regression fits (*R*^2^ ≥ 0.94, *n* = 7 for dose 2 and *n* = 6 for doses 3 and 4).

The disproportionate decreases in AQYs at short wavelengths pose a challenge when quantitatively evaluating the overall impact of photon dose, but a comparison of modeled AQYs at longer wavelengths (≥348 nm), where the exponential regression fit applies, shows statistically significant differences between AQYs at different doses. For wavelengths ≥348 nm, we calculated modeled AQYs in 1-nm increments from the exponential regression equations fit to the experimental AQYs at each photon dose. A plot of the linear regression of natural log AQYs versus wavelength (Fig. 1E) showed highly significant differences between dose 1 and dose 4, and slight but significant differences between other doses at visible wavelengths (i.e., ≥400 nm). Between dose 1 and dose 4, AQYs decreased by a factor of 2 on average across all wavelengths.

### Assessing controls of oil photo-dissolution rates at sea

**Fig. 3.**
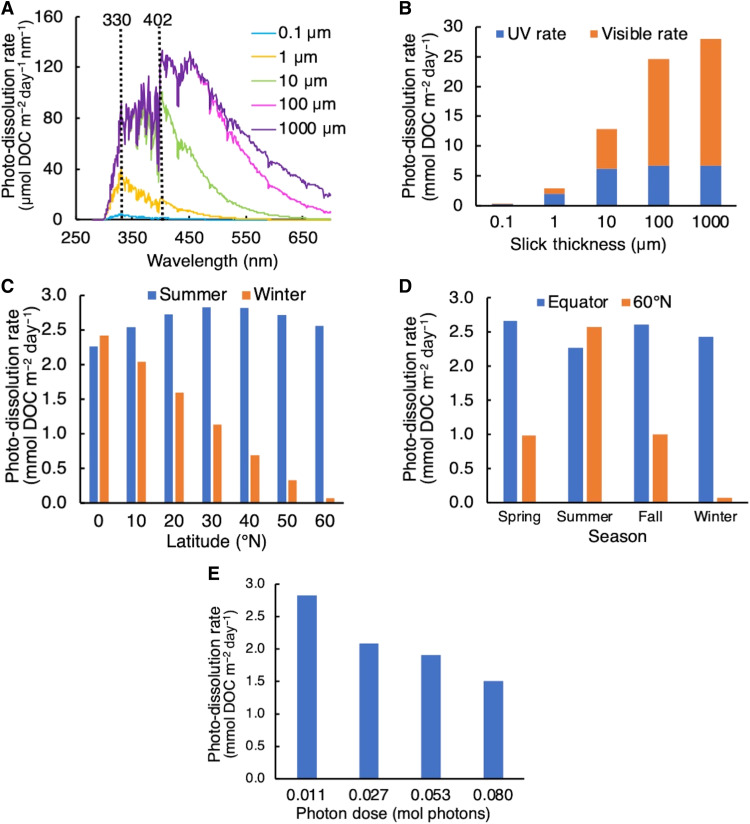
Photo-dissolution rates calculated as a function of environmental variables for sensitivity analysis. (**A**) Action spectra of oil photo-dissolution for slicks of different thicknesses. Dotted black lines show the two peak wavelengths of the AQY spectra, at 330 nm for the slicks ≤1 μm and 402 nm for the slicks ≥10 μm. (**B** to **E**) Total photo-dissolution rate as a function of (B) UV and visible light for varying slick thicknesses (0.1 to 1000 μm), (C) latitude during summer and winter, (D) season at the equator and 60°N, and (E) photon dose. For (A) to (D), rates are calculated from modeled photon dose 1 AQYs determined in this study (Fig. 2A) and hypothetical environmental slick thicknesses and solar irradiances. For (E), rates are calculated from modeled photon doses 1 to 4 AQYs determined in this study (Fig. 2, A to D). Further details on calculations are shown in the “Calculation of photo-dissolution rates for sensitivity analysis” section, section S1, and table S1.

A sensitivity analysis was conducted to determine the relative importance of underlying variables influencing AQYs and light absorption as drivers of overall photochemical rates in a spill scenario (fig. S1 and Fig. 3). The goal of this exercise was to determine which variables are the most important controls on oil photochemistry to guide future research that will facilitate accurate and precise rate calculations in spill models. Photo-dissolution rates were calculated from the modeled AQYs determined in this study (see the previous section, “Calculation of photo-dissolution rates for sensitivity analysis” and section S1) and applied to different hypothetical spill scenarios in which oil slick thickness, time of year (season), latitude, and sunlight exposure time (photon dose) were varied (see the “Calculation of photo-dissolution rates for sensitivity analysis” section). To determine the impact of the chosen wavelength range, rates were also calculated for a spill scenario in which UV light versus UV plus visible light was assumed to initiate photochemistry.

Photo-dissolution rates are highly sensitive to oil slick thickness. Rates for a 100-μm slick are 75 times higher than those for a 0.1-μm slick (Fig. 3, A and B). However, rates only increase by a factor of 1.1 when increasing slick thickness from 100 to 1000 μm, because all UV light and >50% of visible light have been attenuated upon reaching a depth of 100 μm (fig. S2).

Consequently, as oil slicks thicken, the relevant wavelength range of photo-dissolution shifts from UV to visible light. For thin slicks (0.1 to 1 μm), the peak rate occurs in the UV-A region at 330 nm, while for thicker slicks (10 to 1000 μm), the peak rate occurs at 402 nm, the start of the visible region (Fig. 3A). For slicks ≥100 μm, assuming that only UV light contributes to photo-dissolution results in an underestimation of rates by a factor of ~4 (Fig. 2B). Together, slick thickness is major control on both the total oil photo-dissolution rate and the relative importance of UV versus visible light in controlling oxidation.

Seasonal and latitudinal variation in solar irradiance can be major drivers of photo-dissolution rates, but the impact depends on the location and timing of a hypothetical spill. We calculated rates from daily averaged solar irradiance reference spectra in spring (March 20), summer (June 21), fall (September 22), and winter (December 22) over a range of latitudes from the equator to 60°N (*32*). To determine the impact of season, we compared summer and winter rates at each latitude (Fig. 3C). At low latitudes, seasonal variation in irradiance is minor and has little impact on rate: At the equator, summer and winter rates differ by <10%. At high latitudes, seasonal variation can result in rates that are up to ~30 times higher in the summer than in the winter (60°N). To determine the impact of latitude, we compared rates at 60°N to rates at the equator for each season (Fig. 3D). In the spring, summer, and fall months, variation in irradiance across latitudes is minor compared to variation during winter. Irradiance at 60°N is within a factor of 3 of the irradiance at the equator during the spring, summer, and fall, indicating that photo-oxidation at high latitudes is not ruled out as an oil fate process during these months on the basis of available irradiance. Photo-oxidation rates at high latitudes can be as high or higher than rates at the equator during the summer months. In the winter, however, solar irradiance is ~30 times lower at 60°N compared to the equator. Thus, photo-oxidation rates at high latitudes can be as high or higher than rates at the equator during the summer months but are much lower in the winter.

Relative to the other variables tested, photo-dissolution rates are insensitive to photon dose. Rates calculated from AQYs measured at the highest photon dose (dose 4 = 0.080 mol photons) are, on average, a factor of 2 lower than those calculated from AQYs measured at the lowest photon dose (dose 1 = 0.011 mol photons) (Fig. 3E). The photon doses we used in the laboratory corresponded to days to months of exposure to a narrow waveband of sunlight similar in width to that provided by the LEDs (~20 nm) for UV-A and visible light, and months to years of exposure for UV-B light (see the “Modeling photo-dissolution for DwH” section and table S2). Thus, the wavelength-dependent decreases in AQYs determined for these photon doses are similar to what would be expected to occur for the week-long time scales of oil floating on the sea surface and exposed to UV-A and visible light, but are not environmentally relevant for UV-B light (*5*). Therefore, the factor of 2 decrease likely represents a maximum dose-dependent decrease in oil photo-dissolution rates at sea.

### How much DwH surface oil was converted to DOC by photo-dissolution?

**Fig. 4.**
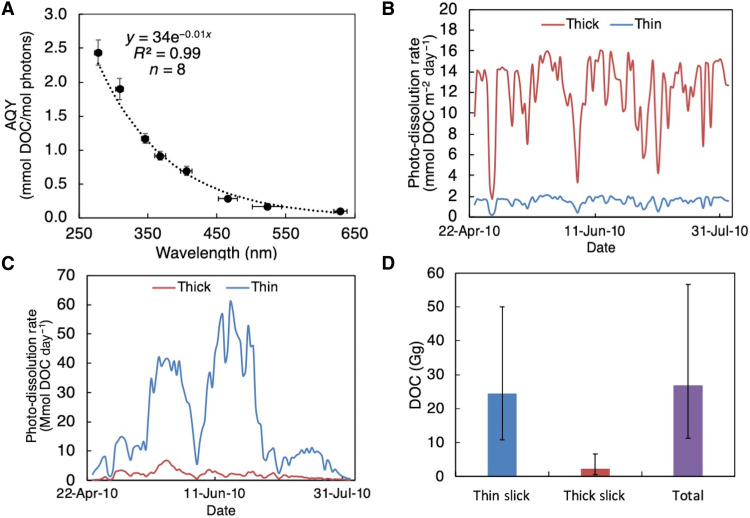
Calculation of DOC produced by photo-dissolution from DwH surface oil. (**A**) AQY spectrum generated from “environmental” AQYs. (**B**) Daily depth-integrated photo-dissolution rates (mmol DOC m^−2^ day^−1^) of the thin (∼1 μm) and thick (∼70 μm) surface slicks throughout the period of surface oiling during DwH in the GoM, calculated from environmental AQYs. (**C**) Daily area-integrated photo-dissolution rates (Mmol DOC day^−1^) for the thin and thick slicks. (**D**) Total photochemical DOC produced throughout DwH. Error bars come from uncertainties in slick areas (*33*) and from uncertainties in environmental AQYs (this study).

To determine the amount of surface oil that may have been subject to photo-dissolution during the 2010 DwH spill, photo-dissolution rates were calculated by combining modeled, environmentally relevant photo-dissolution AQYs (Fig. 4A) with solar irradiance data (*5*) and remote-sensing derived estimates of the surface area and thickness of oil slicks (*33*). Integration of photo-dissolution rates over the 102-day period of surface oiling in the GoM allowed for first estimates of the total mass of DwH surface oil converted to DOC by this process.

Calculating environmentally relevant oil photo-dissolution rates for a spill scenario such as DwH requires AQYs determined for photon doses that correspond to realistic natural sunlight exposure times. For a given photon dose, wavelength-dependent AQYs correspond to different exposure times to natural sunlight because of variation in solar irradiance and the oil absorbance properties (see the “Modeling photo-dissolution for DwH” section and table S2). We selected experimentally determined wavelength-dependent AQYs from photon doses 1 to 4 that corresponded to ~2 to 3 weeks of natural sunlight exposure time in the summer in the GoM for a 70-μm slick, which is the reported average thickness of thick oil slicks generated during the DwH spill (*33*). The spectrum of these “environmental” AQYs demonstrates an exponential decay relationship between AQY and wavelength (*R*^2^ = 0.99, *n* = 8), allowing us to interpolate modeled AQYs in 1-nm increments from the exponential regression equation from 280 to 700 nm (Fig. 4A). For thinner slicks, longer natural sunlight exposure times (approximately months) would be required to achieve the same total light absorption. Because increasing photon dose results in lower AQYs (Fig. 3E), our environmental AQYs are realistic for thick (70 μm) oil slicks persisting in the GoM for weeks after surfacing and conservative (low) for the far more abundant thin oil slicks observed on the sea surface (~98% of total oiled area).

We determined daily photo-dissolution rates for sunlit surface slicks produced by DwH from environmental AQYs, daily solar irradiance on the sea surface during the spill (*5*), and the daily extent of surface oiling (*33*). Calculations were performed for both idealized thin (∼1 μm) and thick (∼70 μm) oil slicks that surface oil was binned into using synthetic aperture radar (*33*). Because of higher rates of light absorption, daily depth-integrated rates (mmol DOC m^−2^ day^−1^) were faster for the thick versus thin slicks (Fig. 4B). However, multiplying the depth-integrated rates by the surface areas of the thin and thick slicks resulted in daily area-integrated rates (Mmol DOC day^−1^) that were substantially higher for the thin slicks because of their greater areal coverage (Fig. 4C). Integration of the daily photo-dissolution rates over the 102-day period of surface oiling (24 April to 3 August 2010) resulted in the production of 24 Gg (range, 11 to 50 Gg) of DOC from the thin slicks and 2 Gg (range, 0.4 to 7 Gg) of DOC from the thick slicks, for a total of 27 Gg of DOC (range, 11 to 57 Gg; Fig. 4D).

## DISCUSSION

### Why does oil photo-dissolution vary with wavelength and photon dose?

Our results provide the first measurement of an AQY spectrum for oil photo-dissolution. We emphasize two characteristics of the spectrum that are relevant for oil fate modeling: the exponential decay in AQYs from the UV to the visible region, and the decrease in AQY values with increasing light absorption (i.e., photon dose dependence).

The exponential shape of the AQY spectrum and the reactivity of oil in the visible region should be accounted for when modeling the fate of oil at sea. The exponential shape of the AQY spectrum observed for a low photon dose validates previous assumptions made in modeling the photochemical fate of oil (*5*), an assumption based on the spectral shape observed for the photo-oxidation of other complex mixtures of organic molecules, such as marine and riverine DOC (*24*–*26*, *34*). Early work on oil photo-oxidation also found that AQYs decreased as a function of wavelength, but lacked sufficient data to determine the spectral relationship (*11*). Despite the decrease in AQY with increasing wavelength, visible light is a major driver of oil photo-dissolution, especially for thick slicks (≥10 μm). The peak wavelength of the action spectrum for slicks ≥10 μm was always 402-nm light, with over >52% of total reactivity driven by visible light (Fig. 3A). The lower AQYs in the visible compared to the UV region (Fig. 2) are overcome by two factors leading to higher rates in the visible compared to the UV region. First, 10 times more visible light than UV light is present in sunlight reaching the Earth’s surface (*32*), and second, visible light penetrates 10 times deeper into oil films than does UV light (fig. S2).

There are three possible explanations for this first report of decreasing oil photo-oxidation AQYs as a function of increased light absorbance (i.e., photon dose dependence). First, oil contains multiple pools of photoreactive compounds with differing intrinsic reactivities. Over time, the most reactive pools are consumed, leaving behind components with lower reactivities. This hypothesis is a common explanation for dose-dependent decreases in AQYs observed for the photo-oxidation of DOC (*26*, *29*, *35*–*37*). A second explanation, which could occur in parallel with the first, is that the dominant reaction mechanism for oil photo-oxidation shifts over time, away from partial photo-oxidation–generating water-soluble compounds and toward complete photo-oxidation–generating CO_2_. Competition between reaction pathways could also explain the unequal impact of photon dose across wavelengths that we observed, as certain pathways may be favored at particular wavelengths. For example, AQYs for complete photo-oxidation of DOC to CO_2_ decrease exponentially from the UV-B to the visible region (*25*), consistent with disproportionate sensitivity to photon dose in the UV versus visible region in our experiments (Fig. 2). Third, if the initial rates of oxidation are fast enough that they outpace rates of oxygen diffusion into the oil, photo-oxidation may become O_2_ limited. In this situation, the intrinsic reactivity of the oil remains the same, but O_2_ limitation decreases the apparent rates of reactivity. We found that O_2_ limitation does not explain the reduced AQYs in our experimental system, as shown by the fact that AQYs decreased in response to increasing photon dose rather than increased incident irradiance (and therefore increased oxidation rate), but we cannot rule it out in other environmental scenarios (sections S2 and S3).

### What variables control photo-dissolution in a spill scenario?

The sensitivity analysis reveals that several variables influencing the amount of light absorbed by oil are key controls on photo-oxidation rates. Specifically, variation in oil slick thickness, along with seasonal and latitudinal variation in solar irradiance, can result in changes in oil photo-dissolution rates by up to two orders of magnitude. While this analysis does not account for all factors that influence photo-oxidation at sea (see section S3), it represents a major advance in our capacity to assess the relative importance of photochemical weathering in past and future spill scenarios.

The sensitivity of oil photo-dissolution rates to slick thickness points to the importance of advancing remote sensing tools for the analysis of oil slick thickness and surface area at sea. Slick thickness was the most important control on the overall photo-dissolution rate, resulting in variation in rates by one to two orders of magnitude over a range of thicknesses realistic for a spill scenario (0.1 to 1000 μm). Slick thickness also controls the relative contributions of UV versus visible light to oil photo-dissolution: Visible light accounts for just over a quarter (28%) of photo-dissolution for thin (0.1 μm) slicks but three-quarters (76%) for thick (1000 μm) slicks. Thus, accurate prediction of photo-oxidation rates in future spills will rely on accurate and high-precision information about the extent and thickness of floating oil. Advances in remote sensing tools such as synthetic aperture radar were critical to determining the fate of DwH oil (*33*), but accurate and precise analysis of thick slicks and, in particular, emulsions is still an area of active research (*38*). The development of next-generation remote sensing tools for rapid, accurate, and precise analysis of oil film surface area and thickness is thus critical for improving forecasts of oil photo-oxidation rates.

The sensitivity of oil photo-dissolution rates to latitudinal and seasonal variation in solar irradiance reveals the importance of oil spill location and timing when evaluating the role of photochemistry in oil weathering. Much previous work has assumed that oil photo-oxidation is primarily relevant for spills in low-latitude areas with high solar irradiance year-round (*39*). An evaluation of solar irradiance as a function of latitude and season reveals a more complex picture: Solar irradiance at 60°N in the summer is comparable to solar irradiance at the equator at the same time of year (*32*); thus, photo-oxidation could be just as relevant at high latitudes as in lower latitude waters (e.g., GoM) depending on the timing of a spill.

This finding raises the possibility that photo-oxidation may be a relevant fate process for oil spilled in the Arctic Ocean and northern latitude waters, where the likelihood of spills is increasing because of ship traffic in the increasingly ice-free Northwest Passage and Northern Sea Route (*40*, *41*). Photo-oxidation was invoked as a potential explanation for the overestimation of petroleum hydrocarbons in the water column by fate models of the 1989 *Exxon Valdez* spill in Prince William Sound, Alaska (*17*). While the oxygen content of *Exxon Valdez* surface and stranded oil was not measured, these findings highlight the need to consider photo-oxidation in future spills in high-latitude waters.

Our analysis does not cover all factors that may influence oil photo-dissolution rates both positively and negatively, such as sky conditions, O_2_ limitation, oil type, and temperature (fig. S1), all of which are discussed in section S3 and should be systematically investigated in future research. Differences between our experimental system and the ocean may also result in differences in rates between the laboratory and at sea. Most notably, we irradiated oil samples on smooth glass disks in the absence of seawater, which was necessary to control film thickness, the dominant driver of oxidation rates (Fig. 3, A and B). If seawater were present during the irradiation, then it may have increased photo-dissolution rates (section S3).

### How does photo-dissolution compare to other environmental fates of DwH surface oil?

**Fig. 5.**
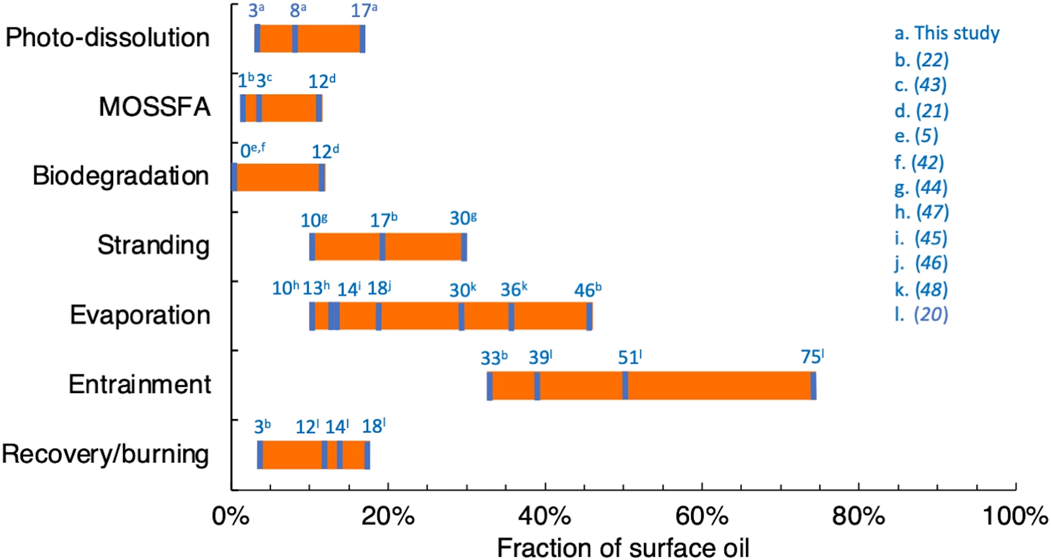
Fractions of DwH surface oil attributed to various fate processes. The orange bars represent the full range of values presented in the literature. Where individual studies give a single value, or where the same study gives minimum, maximum, and best estimate values, they are marked. Note that some values may differ from those presented in the original study because they were recalculated as a fraction of the total cumulative surface oil over the course of the DwH spill. Also note that not all of the fate processes shown are ultimate fates that are independent of one another. For example, entrained oil may subsequently be degraded biologically or photochemically, or may contribute to MOSSFA. Thus, there is no expectation that the percentages shown here add up to 100%.

Our first estimates suggest that the amount of DwH surface oil photo-oxidized to DOC is comparable in magnitude to other widely recognized fate processes (e.g., evaporation and stranding on coastlines; Fig. 5), thereby inviting a critical reevaluation of surface oil budgets for both DwH and future spills. We estimated that 27 Gg (range, 11 to 57 Gg) of DOC were produced by sunlight throughout the 102-day period of DwH surface oiling (Fig. 4D). Given that the total cumulative mass of hydrocarbons that arrived at the sea surface was ~372 Gg (87% C by mass; see section S4), 3 to 17% of the surface oil may have been transformed by sunlight into DOC, with a best estimate of 8%. This fraction is comparable to other oil fate processes, but, because kinetic information for photo-dissolution was previously unavailable, no oil budget has ever included it as a mass balance term for any spill. For DwH, a review of the fractions of surface oil apportioned to various fate processes in the literature shows that photo-dissolution may have removed as much or more oil from surface waters (3 to 17% by mass) as marine oil snow [MOSSFA (marine oil snow sedimentation and flocculent accumulation), 1 to 12%], biodegradation (0 to 12%), and mechanical recovery/burning (3 to 18%), and is comparable in magnitude to stranding (10 to 30%) and evaporation (10 to 46%) (Fig. 5 and section S4) (*5*, *20*–*22*, *42*–*48*). Thus, inclusion of photo-dissolution in the DwH oil budget may help spill scientists balance the books for the fate of DwH surface oil (*21*).

Our estimate of oil photo-dissolution is likely conservative because it only includes dissolution of oil in floating slicks, neglecting potential contributions from oil entrained in surface waters. Entrainment, consisting of oil dispersed into the 40-m GoM surface mixed layer (*22*) by both natural dispersion and chemical dispersants, was by far the most important fate process for DwH surface oil, affecting 33 to 75% of the oil (*20*, *22*). A value at the lower range of these variable estimates is more likely because these estimates do not take into account the reduced effectiveness of chemical dispersants due to photo-oxidation (*6*). Moreover, some fraction of this entrained oil was likely susceptible to and removed by photo-dissolution. For example, the wavelength of light at the peak of the action spectrum for thick oil (402 nm; Fig. 3A) penetrates throughout the entire 40-m-deep mixed layer (section S5). These findings highlight the important ramifications of incorporating photo-dissolution into oil fate models, affording refined estimates of the magnitude of other surface oil fate processes (Fig. 5).

### Environmental implications of photo-dissolution for future oil spills

Our findings on the photo-dissolution of oil at sea have notable implications for the oil spill modeling and response community. Our results call for and provide key pieces of empirical data needed to account for photo-dissolution in oil spill transport and fate models. Currently, no spill models include photo-dissolution (*2*). Our sensitivity analysis provides the first assessment of the factors most important to consider when modeling photo-dissolution at sea, finding that rates are most sensitive to slick thickness, season/latitude, and wavelength and less sensitive to photon dose. Our finding that photo-dissolution may have accounted for the removal of a substantial fraction of DwH oil invites a reevaluation of the guidance stated in *Oil in the Sea III* that photo-oxidation does not affect oil mass balance (*19*). Incorporation of photo-oxidation into Lagrangian oil fate models for future spills will enable more accurate estimates of the location and extent of surface oiling and refinement of estimates for other fate processes, collectively affording a more judicious allocation of resources during the response phase. For example, an increase in the fraction of oil expected to photochemically dissolve in water may be beneficial in reducing the load of material that responders must plan to burn, skim, and chemically disperse at sea or physically remove from sensitive coastal ecosystems.

On the other hand, the ecosystem impacts of water-soluble photo-products on marine microbial communities must also be considered by response scientists and natural resource damage assessments. The 27 Gg of DOC added to offshore GoM waters during DwH is a small fraction (~4%) of the amount of DOC exported from the Mississippi River to the GoM during the spring and summer (675 Gg; section S6). However, this oil-derived DOC is presumably chemically distinct from terrestrial and autochthonous marine DOC (*49*) and is presumably more bioavailable than the insoluble slick oil (*50*, *51*). Consistently, previous work has reported that oil photo-products can stimulate microbial activity (*52*, *53*), suggesting that water-soluble oil photo-products may exhibit low persistence in the ocean. However, it is unlikely that all oil photo-dissolution products are readily respired by microbes. DOC concentration and fluorescence anomalies, presumably driven in part by photo-dissolution, persisted in GoM surface waters for up to 3 years after the DwH spill (*49*). To date, only a few studies have characterized the toxicity of oil photo-products to aquatic life (*54*–*56*), finding mixed results. While the results of this study suggest that photo-dissolution was a major fate of DwH surface oil, the net balance of the potential impacts of oil photo-dissolution on ecosystem health remains an open question that should be explored in future research.

## MATERIALS AND METHODS

### Sample collection and preparation

Crude oil (SO-20100818-HP1-55072-001) from the Macondo well in the GoM, the source of the 2010 DwH oil spill, was collected by *Helix Producer I* in August 2010. To remove oil components that evaporated to the atmosphere or dissolved in water and thus were not present in the floating surface slicks (<~n-C_14_), we weathered the oil in the dark at 53°C until 35% by mass of the oil had evaporated (*45*). Previous work showed that Macondo oil weathered according to this protocol resulted in a hydrocarbon composition similar to that of the insoluble, nonvolatile oil present in surface slicks and exposed to sunlight during the DwH spill (*5*). The oil was stored in the dark at room temperature before experimentation.

Seawater was collected from Martha’s Vineyard Sound in Falmouth, MA using precombusted 4-liter glass bottles (450°C for 4 hours). To remove particulates, seawater was filtered through a precombusted 0.7-μm pore size GF/F glass fiber filter (Whatman) and a 0.22-μm pore size polyethersulfone filter (Sterivex) and then poisoned with 0.1% (v/v) of a saturated HgCl_2_ solution (Acros Organics). Separate seawater samples were collected for each photon dose experiment (described below) and were stored in precombusted glass bottles in the dark at 5°C until further use.

### Photochemistry experiments

We irradiated oil using a custom-built LED reactor assembly (Fig. 1) (*24*). Briefly, for each reactor, an oil film of uniform thickness (200 μm) was prepared on a smooth glass disk (McMaster-Carr) using a 350-mg aliquot of 35% evaporated Macondo oil. The thickness of the slick was determined from the total volume of oil added to the disk and the surface area of the disk. The sample was positioned at a predetermined height on an aluminum platform (inner housing). The oil film was then covered by an aluminum outer housing (painted black matte to minimize light scattering) on which a high-powered (≥100 mW; LG 6060 series) LED chip that provided a narrow waveband of light (~20 nm) directed toward the oil sample was affixed. A similar version of the reactor assembly was validated for use in determining AQY spectra of DOC photochemical oxygen consumption using LED chips at five wavelengths (278, 309, 347, 369, and 406 nm) (*24*). For this study, we added three more wavelengths in the visible region (466, 526, and 629 nm). The eight wavebands were chosen such that the spectral outputs from each LED had minimal overlap (Fig. 1C). Our system allowed us to achieve equivalent photon doses, or equal numbers of moles of photons absorbed, by the oil samples across all wavelengths (section S2). All samples were irradiated alongside a dark control.

We irradiated oil at four different photon doses (doses 1 to 4), each corresponding to a different sunlight exposure time for an oil slick on the sea surface in the GoM (fig. S3). We ensured that differences in AQYs calculated for doses 1 to 4 were due to changes in the photon dose (total number of moles of photons absorbed) and not a related but distinct variable, irradiance (moles of photons delivered per area per time), by manipulating the photon dose via different methods (section S2). For each experimental trial, the photon doses for all LEDs were within 15% of the target value (fig. S3). The approximate amount of time it would take oil to absorb each experimental photon dose from discrete wavebands of UV-B, UV-A, and visible light present in natural sunlight in the GoM was determined (see the “Modeling photo-dissolution for DwH” section). The temperature of the samples under the LEDs was 26° ± 2°C, as measured by pointing an infrared thermometer directly at the oil immediately after stopping each irradiation. This is within the range of surface oil temperatures measured in the GoM during DwH (*57*).

### Dissolution and DOC analysis

After irradiation, the oil was equilibrated with the Hg-poisoned, filtered seawater (125 ml) in the dark, on a shaker table for 24 hours at 30°C and 100 rpm. To remove the water-insoluble oil, each leachate was filtered through a Milli-Q–rinsed, 0.22-μm Sterivex filter into three separate precombusted glass 40-ml vials (Thermo Fisher Scientific). Samples were acidified with trace metal–grade HCl, and DOC was quantified as CO_2_ after high-temperature combustion using the Shimadzu 5000A TOC Analyzer (*58*). To test whether DOC was saturated in our experimental system, we determined DOC production as a function of oil-to-water ratio and found that it was not limited by saturation (section S7 and fig. S4). To test whether biodegradation could have contributed to DOC production, we monitored how two well-established indicators of biological oxidation, the heptadecane (n-C_17_)–to–pristane ratio and the octadecane (n-C_18_)–to–phytane ratio (*59*), changed in the dark throughout the longest exposure period (7.5 days). Stable n-C_17_/pristane and n-C_18_/phytane ratios throughout the exposure period indicate that biological activity did not contribute to DOC production in our experimental system (section S8).

### Calculation of photo-dissolution rates for sensitivity analysis

Photo-dissolution rates determined for the sensitivity analysis were calculated following Eq. 1

$$\text{Photo}-\text{dissolution rate}\;(\text{mol}\;C\;m^{-2}\;{\text{day}}^{-1})=\int_{280}^{700}\Phi_{\lambda }R_{a,\lambda }\;d\lambda$$ (1)

where Φ_λ_ is the wavelength-dependent AQY (mol product per mol photons absorbed), *R*_a, λ_ is the wavelength-dependent rate of light absorption (mol photons absorbed m^−2^ day^−1^ nm^−1^), and λ is wavelength (nm), integrated from 280 to 700 nm. For all tests in the sensitivity analysis besides the photon dose test, AQYs used to calculate rates were interpolated as a function of wavelength from the photon dose 1 AQYs determined from the experimental, 200-μm-thick oil films in laboratory (Fig. 2A). For the photon dose test, the AQYs used to calculate rates were interpolated in 1-nm increments as a function of wavelength from the experimental AQYs from either dose 1, 2, 3, or 4 (section S1). For the wavelength range test, the limits of integration were changed to 280 to 400 nm or 400 to 700 nm to account for the differing contributions of UV and visible light, respectively, to the overall rate.

The rate of light absorption by oil, *R*_a, λ_, was calculated by Eq. 2

$$R_{a,\lambda }\;(\text{mol}\;\text{photons absorbed}\;m^{-2}\;{\text{day}}^{-1}\;{\text{nm}}^{-1})=E_{\mathrm{o\lambda}}(1-e^{-a_{\lambda }z})$$ (2)

where *E*_oλ_ is the downwelling spectral photon irradiance to the sea surface (mol photons m^−2^ day^−1^ nm^−1^), *a*_λ_ is the wavelength-dependent attenuation coefficient of the oil (m^−1^), and *z* is the oil slick thickness (m). For the slick thickness test, light absorption rates were calculated for a range of hypothetical oil slick thicknesses (0.1 to 1000 μm) representative of the range detected on the sea surface by remote sensing in a spill scenario (*33*). For the seasonality and latitude dependence tests, rates were calculated from solar irradiance for all four seasons (March 20, June 21, September 22, and December 22) and as a function of latitude for every 10° from 0°N to 60°N from daily 24-hour averaged reference spectra (*32*). The specific conditions used in each test are shown in table S1.

### Modeling photo-dissolution for DwH

To determine the importance of photo-dissolution as a fate process for DwH surface oil, daily photo-dissolution rates for DwH were determined using Eq. 1. Modeled environmental AQYs (Fig. 4 and table S2) that correspond to environmentally relevant exposure times for floating oil during the spill were inserted for Φ_λ_. Light absorption was determined using Eq. 2 from experimentally determined Macondo oil absorption coefficients and field data on irradiance and slick thickness throughout the course of the spill.

The environmental AQYs were determined from our experimental AQYs as follows: We determined the approximate amount of time it would take oil to absorb each experimental photon dose from discrete wavebands of natural sunlight in the GoM using Eq. 3 (table S2)

$$t=\frac{Q}{S*E_{o}*(1-e^{-az})}$$ (3)

where *t* is the environmental exposure time; *Q* is the laboratory photon dose (mol photons); *E*_o_ is the integrated solar irradiance over a discrete, 20-nm waveband of light centered around the lambda max of the LED; *a* is the average oil absorption coefficient over that 20-nm waveband; and *z* is the oil slick thickness. We completed this calculation for light absorption from 20-nm wavebands of sunlight, which is similar in width to the spectral output of the LEDs (average full width at half maximum = 17 nm), in increments from 280 to 700 nm. We assumed a thick 70-μm oil slick (the reported average thickness of thick oil slicks generated during the DwH spill) (*33*) with the same surface area as our experimental samples (0.001963 m^2^) and determined solar irradiance from a daily averaged, clear-sky summer 30°N reference spectrum (*27*). We note that Eq. 3 is not strictly correct but provides an estimate of exposure time where *E*_o_ and *a* do not vary much over a 20-nm waveband of natural sunlight. The advantage of this calculation is that it tells us the environmental exposure time that each experimental AQY, under a 20-nm LED, is directly comparable to. We selected wavelength-dependent AQYs from photon doses 1 to 4 that corresponded to ~2 to 3 weeks of natural sunlight exposure time in the summer in the GoM for a 70-μm slick. For thinner slicks, the approximate exposure times required to achieve the same photon dose would be longer. Thus, our estimates of the amount of time it would take natural sunlight to result in the laboratory photon doses are conservative. As a result, we choose conservative AQYs (higher photon doses correspond to lower AQYs) as our environmental AQYs and err on the side of a conservative calculation for the extent of photo-dissolution during DwH.

Irradiance throughout the period of surface oiling was determined as described previously (*3*) from daily fluxes of UV-B, UV-A, and PAR (photosynthetically active radiation) light that were measured from 24 April to 3 August 2010 at the Louisiana State University Central Research Station as part of the U.S. Department of Agriculture UVB Monitoring Climatological and Research Network (30.36 N, 91.17 W). Oil slick thickness was categorized as either thin and “sheen-like” or thick and “emulsion-like,” where the thin slicks were approximated as 1 μm and the thick slicks as 70 μm from analysis of synthetic aperture radar images taken during the spill (*28*).

Total DOC produced during the DwH spill was calculated by multiplying rates by the total areas of the thin and thick slicks on each day of surface oiling and then integrating over the 102-day period of surface oiling. Uncertainty in the DOC estimate was determined from the SDs in environmental AQYs and SDs in surface slick areas (i.e., ±5028 km^2^ for the thin film and ±145 km^2^ for the thick film) (*28*). The contributions of the uncertainties in the AQYs and the slick areas to the overall uncertainty in the DOC estimate are similar in magnitude. To determine the fraction of surface oil lost as DOC, the total cumulative mass of hydrocarbons that arrived at the sea surface over the 102-day period of surface oiling was calculated and compared to the total cumulative mass of DOC produced, assuming that surface oil was 87% C by mass (section S4) (*60*).

## Supplementary Materials

### This PDF file includes:

Supplementary Text Figs. S1 to S4 Tables S1 to S4 References
