# Supplementary material for: Sunlight-driven dissolution is a major fate of oil at sea
Source: Sci Adv. 2022 Feb 16;8(7):eabl7605. doi: 10.1126/sciadv.abl7605 (PMC8849300; doi:10.1126/sciadv.abl7605)
Supplement: Supplementary file 1 — Supplementary Text Figs. S1 to S4 Tables S1 to S4 References [file sciadv.abl7605_sm.pdf]

Science Advances
AAAS

Supplementary Materials for

Sunlight-driven dissolution is a major fate of oil at sea

Danielle Haas Freeman and Collin P. Ward*

*Corresponding author. Email: cward@whoi.edu

Published 16 February 2022, *Sci. Adv.* **8**, eabl7605 (2022)
DOI: 10.1126/sciadv.abl7605

This PDF file includes:

Supplementary Text
Figs. S1 to S4
Tables S1 to S4
References

Supplementary Text

Appendix S1: Interpolation of modelled AQYs from experimental AQYs for photo-dissolution rate calculations in sensitivity analysis

Modelled AQYs were determined in one-nanometer increments from 280-700 nm in order to calculate oil photo-dissolution rates in Eqn 1. Modelled AQYs were determined from the eight experimentally determined AQYs (278, 309, 347, 369, 406, 466, 526, 629 nm) at each of the four photon doses. For Dose 1, all eight experimental AQYs fell onto the exponential regression line (R^2 = 0.98), so modelled AQYs were interpolated from the exponential regression equation for 280-700 nm (Figure 2A). For Dose 2, seven experimental AQYs (≥ 309 nm) fell onto the exponential regression (R^2 = 0.99), so modelled AQYs for wavelengths ≥ 309 nm were interpolated from the exponential regression equation (Figure 2B). Modelled AQYs for 280-308 nm were set equal to the experimental AQY which fell off of the exponential regression curve (277 nm, AQY = 1.396 mmol DOC per mol photons). For Doses 3 and 4, six experimental AQYs (≥ 346 nm) fell onto the exponential regression (R^2 = 0.99), so modelled AQYs for wavelengths ≥ 346 nm were interpolated from the exponential regression equation (Figure 2C-D). Modelled AQYs for 280-345 nm were interpolated from a linear regression between the two experimental AQYs (277 and 309 nm) which fell off of the exponential curve (Figure 2C-D).

Appendix S2: Controlling and calculating photon dose using LED photoreactors

We controlled the photon dose in our experimental system by making three adjustments: First, we adjusted the total irradiant output of the LEDs, which were powered by separate, tunable power supplies. Second, we adjusted the distance between the sample and the LED (4-14 cm) by changing the height of the aluminum platform, an adjustment that also altered the incident

irradiance seen by the sample. Lastly, we adjusted the irradiation exposure time. The lower-powered (100 mW) short-wavelength LEDs (278-347 nm) required a longer irradiation time (5-7.5 days) to achieve the same photon dose as the higher-powered (2-6 W) long-wavelength LEDs (369-629 nm), which had irradiation times of 1-1.5 days.

To account for the possibility that decreases in AQYs we observed at higher photon doses could be due to increased irradiance (and therefore increased oxidation rate), rather than an increase in the total moles of photons absorbed, we employed the adjustments discussed above in separate experimental trials. Between Doses 1-3, we achieved the desired dose by altering the irradiance that the sample was exposed to. Between Doses 3-4, we maintained the same irradiance and achieved a higher photon dose by lengthening the exposure time. Photo-dissolution AQYs decreased significantly between photon doses 3 and 4 (see Results), indicating that these decreases cannot be attributed to differences in irradiance levels between experiments, and are rather due to differences in the total photon dose.

Experimental photon doses were calculated by measuring the irradiant output under the LEDs at five positions under location of the sample using a NIST-calibrated spectroradiometer (StellarNet, Black Comet C-50). For each sample, the irradiances were averaged and then converted to mols photons absorbed by Eqn S1:

$$Q_{a,\lambda} = \int_{\lambda_{min}}^{\lambda_{max}} E_{o,\lambda} S(1 - e^{-a_\lambda * z})\, t\, d\lambda \qquad \textit{Eqn S1}$$

where $Q_{a,\lambda}$ is the moles of photons absorbed, $E_{o,\lambda}$ is the irradiance output by the LED (mols photons m^{-2} s^{-1} nm^{-1}), S is the surface area of the oil film (m^2), a_λ is the attenuation coefficient of the oil (m^{-1}), z is the film thickness (m), t is the experimental exposure time (s), and λ is wavelength (m). Oil attenuation coefficients were determined by measuring absorbance of a 1200x diluted oil solution in gas-chromatography grade methylene chloride (Fisher Scientific) on a spectrophotometer (Perkin Elmer Lambda 650s; (*5*)).

Spectral reflectance off of the glass disks had a negligible impact on the photon dose seen by the oil sample. For all wavelengths, > 99% of light was absorbed by the 200-µm thick oil samples, except for the 629 nm LED, in which case ~ 80% of light was absorbed (Fig. S2). Spectral reflectance off the glass disks was approximately 7% from 615 to 645 nm (FWHM range of the 630 LED). The penetration of 20% of the 629 nm light through the sample resulted in < 2% back-reflection off the glass disk, resulting in a negligible increase in the moles of photons absorbed by the sample.

<u>Appendix S3: Factors not addressed in the photo-dissolution rate sensitivity analysis</u>

Several factors not addressed in our sensitivity analysis may impact rates of photo-oxidation at sea. Specifically, sky conditions that alter incident irradiance at the sea surface and possible O_2 depletion in oil slicks could both decrease photo-dissolution rates in spill scenarios relative to the rates calculated in this study. Colder temperatures at high latitudes could also decrease rates. Conversely, the presence of seawater could increase photo-dissolution rates. Differences in oil type may increase or decrease rates. These variables are discussed in more detail below.

The solar irradiance spectra used to calculate rates in the sensitivity analysis (i.e., not the DwH calculations described in Section 4.5 of the main text) assumed clear sky conditions. Cloudiness would decrease the total irradiance that oil is exposed to, but incident irradiance and sky conditions are rarely measured in during marine spills. For example, during the DwH spill, the community relied on irradiance monitoring data from the USDA UV monitoring station in Baton Rouge, LA (*5*) and the EPA UV and Ozone Network station in Houston, TX (*63*). These stations are 330 and 690 km from the DWH wellhead, respectively, likely introducing a source of uncertainty to oil photo-oxidation rates and photo-toxicity to aquatic animals. Given the diverse roles that sunlight plays in oil spill science (e.g., fate and transport, alteration to physical and chemical properties, toxicity, and oil spill response operations (*2*)), expansion of irradiance monitoring on oil tankers and offshore oil fields would substantially improve assessments of the fate and impacts of oil spilled at sea.

Oxygen depletion in oil slicks could decrease rates of photo-oxidation at sea. We found that O_2 depletion was not a factor influencing rates in our experimental system (see in Section 3.1 in main text and Appendix S2). Moreover, given that the irradiances used in our experiments were 1- to 14-fold greater than natural sunlight, we do not expect that O_2 limitation occurs for the relatively fresh, thin films of light crude oils like Macondo oil. However, we cannot rule out this factor for heavier, more viscous crude oils or weathered oils (e.g., emulsions) with lower O_2 diffusion rates (*64*). Future research should determine the extent in which emulsification impacts photo-oxidation rates.

Colder temperatures at high latitudes could result in a decrease in photo-dissolution rates. In this study, oil samples were irradiated at 26°C and then equilibrated with seawater at 30°C. These temperatures were typical of GoM seawater temperatures during the summer of 2010 when the DwH spill occurred *(57)*. However, Arctic summer seawater temperatures are ≤ 10°C *(61)*. Lower temperatures may decrease photo-dissolution rates, both by decreasing the rate of photo-oxidation reactions and by decreasing the solubility of oil photo-products. Temperature is expected to have only a minor impact on photo-oxidation reaction rates, with a 10°C decrease in temperature typically decreasing reaction rate by less than a factor of 1.5 *(58*, *62)*. However, the water-solubility of crude oil photo-products has not been investigated as a function of temperature, so it is conceivable that lower water-solubility at high latitudes could decrease photo-dissolution rates compared to what is shown in our sensitivity analysis.

Finally, our sensitivity analysis does not account for differences in photo-reactivity across oil types. Oils vary greatly in chemical composition, including in the aromatic, resin, and asphaltene components that absorb sunlight and can initiate photochemistry. For example, the percentage of aromatics can range from 5% in a diesel fuel to 50% in a Bunker C fuel oil (*64*). Moreover, the concentration of sulfur-containing compounds, which are known to be highly photoreactive (*12*), can vary from 0-5% of crude oil composition from sweet to sour oils (*64*). Laboratory studies have shown that the oil photo-oxidation rates can range widely depending on oil type, but it is currently unknown how or if chemical composition drives reactivity (*3,65*), a gap in knowledge that should be systematically evaluated in future research.

Differences between our experimental system and the ocean may also result in differences in rates between the laboratory and at sea. Most notably, we irradiated oil samples on smooth glass disks in the absence of seawater, which was necessary to control film thickness, the dominant driver of oxidation rates (Fig. 3A-B). If seawater were present during the irradiation, it may have increased photo-dissolution rates in two ways. First, water could have provided an additional source of oxygen to be incorporated into oil. Our previous findings demonstrated that the photochemical addition of oxygen from water was at most 15% of total oxidation (*66*), suggesting that the rates reported in this study could be up to 15% lower than expected at sea. Second, the presence of water could have facilitated progressive dissolution over time, preventing any buildup of water-soluble products on the surface of the oil and allowing for greater exposure of the underlying oil to further oxidation. The extent to which progressive dissolution would have impacted our findings is not known and is extremely challenging to test given the dynamic nature of oil film thickness on seawater.

Appendix S4: Calculation of DwH surface slick mass and fate

The total cumulative mass of hydrocarbons that arrived at the sea surface over the 102-day period of surface oiling was calculated from the total volume of hydrocarbons spilled (5 million barrels (*67*)) and the fraction of total hydrocarbons which surfaced (43% (*21*)). The density of the oil was assumed to be 132.2 kg liquid oil per barrel with a (total hydrocarbons)/oil ratio of 1.31 (*45*). This ratio accounts for the fact that liquid oil (≥ C5) made up only a fraction of the total material released, which included natural gas (C1-C4). For this study, the mass of surfacing material was calculated in terms of the total mass of material released, referred to as "hydrocarbons," including both oil and gas. The vast majority (> 95%) of the natural gas

remained in the deep plume and did not surface, but small fractions of these volatile compounds contributed to the evaporative flux out of surface slicks (*46*). After evaporation, the remaining hydrocarbons in the surface slicks were all "oil"—insoluble, nonvolatile compounds, which is why the material present in the surface is colloquially referred to as "surface oil" in the main text. The slicks were assumed to be 87% C by mass (*59*).

The fractions of surface hydrocarbons which disappeared from the sea surface by various fate processes were collected from the literature (Lehr et al., 2010 (*20*); Ryerson et al., 2012 (*45*); Boufadel et al., 2014 (*44*); Stout et al., 2016 *(42)*; Passow and Hetland 2016 (*21*); Joye et al., 2016 (*47*); Gros et al., 2017 (*46*); Stout and German, 2018 (*43*); Ward et al., 2018 (*5*); Kujawinski et al., 2020 (*48*); and French McCay et al., 2021 (*22*)) and are presented in Fig. 5 in the main text. Some values presented in Fig. 5 appear to differ from the values presented in the original studies for many reasons, including: (i) the original values were calculated as a fraction of the total oil released, before or after accounting for direct recovery at the wellhead, (ii) some studies assume that all natural gas (defined as ≤ C5) compounds remained subsurface while others assume that a small fraction of natural gas compounds surfaced and contributed to the evaporative flux, (iii) some studies express their percentages in terms of total liquid oil while others express their percentages in terms of total hydrocarbons or "petrocarbon" (including the natural gas flux from the well), and (iv) some studies express their percentages in terms of surface oil (or hydrocarbons/petrocarbon) only, but vary in terms of the fraction of released material that they assume arrived at the surface. An explanation of the original values shown in the studies referred to in Fig. 5 is shown in Table S3.

The recalculated values shown in Fig. 5 were determined as follows: French-McCay et al. (*22*), Gros et al. (*46*), Passow and Hetland (*21*), Joye et al. (*47*), and Kujawinski et al. (*48*) provide independent estimates of the cumulative mass of hydrocarbons which arrived at the sea surface (57% of total hydrocarbons released before direct recovery from French-McCay et al. (*22*), 60% from Gros et al. (*46*), 43% from Passow and Hetland (*21*), and 39-50% from Joye et al. (*47*)). Based on their own estimates for the fraction surfacing hydrocarbons, we calculated the mass of surface hydrocarbons arriving at the sea surface. We then recalculated the values they present for the percentages of total hydrocarbons or total oil removed by evaporation, entrainment, burning/recovery, and MOSSFA as a fraction of the surfacing hydrocarbon mass. The French-McCay et al. (*22*) and Joye et al. (*47*) studies required the further assumption that all surfacing material was oil, with no contributions from natural gas, a fair assumption given that the vast majority (> 95%) of natural gas compounds remained in the deep plume (*46*). For Lehr et al. (*20*) we recalculated the percentages of material removed by natural dispersion, chemical dispersion, burning, and skimming, as a fraction of the total surfacing hydrocarbon mass, assuming (1) 43% of the total hydrocarbon mass surfaced, as reported by Passow and Hetland (*21*) and (2) all surfacing material was oil. For Stout and German (*43*), we calculated the percentage of material removed by MOSSFA as a fraction of the total surfacing hydrocarbon mass, again assuming that 43% of the total hydrocarbon mass surfaced. Boufadel et al. (*44*) and Ryerson et al. (*45*) presented their percentages of oil stranded (*44*) and evaporated (*45*) as a fraction of total surfacing hydrocarbons, so no adjustments were necessary for these values. For Kujawinski et al. (*48*), masses of cumulative nonvolatile, insoluble surface oil, as well as masses of volatile surfacing hydrocarbons, were presented. We calculated the fraction of evaporated hydrocarbons from these values.

Appendix S5: Calculation of light attenuation in GoM surface waters

In order to evaluate whether oil entrained in the GoM surface mixed layer could be susceptible to photo-oxidation, we calculated the fraction of 402-nm light that had not yet been attenuated at the bottom of the 40-m mixed layer. We chose to calculate the attenuation of 402-nm light because this is the peak of the action spectrum for the photo-dissolution of thick (>10 μm) oil (Fig. 3A). In order to determine the attenuation coefficient (K_d) of 402-nm light in the GoM, we assumed that attenuation was driven by CDOM, a fair assumption given that ocean CDOM drives upwards of 90% of UV and violet light attenuation (*68*). We calculated a K_d from the spectral slope and 320-nm attenuation coefficients of CDOM spectra from several GoM summer samples according the relationship presented in Powers and Miller (*37*):

$$K_d \sim a_{402\,nm} = a_{320\,nm} e^{-S_{ag*(402-320)}} \qquad \text{Eqn S2}$$

Where a is the CDOM attenuation coefficient (approximately equal to GoM seawater K_d) at either 402 or 320 nm and S_{ag} is the spectral slope. We calculated the percentage of 402-nm light remaining at the 40-m mixed layer depth as:

$$\frac{E_z}{E_o} = e^{-K_d * z} \qquad \text{Eqn S3}$$

Where E_z/E_o is the ratio of solar irradiance at depth z to the irradiance at the surface (E_o). We found that 8% of 402-nm light was still available at 40 m and light at this wavelength was not completely attenuated (< 1% available) until 70 m of depth, indicating that this light at the peak of the photo-dissolution action spectrum was available all throughout the mixed layer. Blue wavebands (450–490 nm), which have lower CDOM attenuation coefficients and are capable of oxidizing oil would, in theory, be present at higher levels throughout the mixed layer than the 402 nm, violet waveband presented in this calculation.

Appendix S6: Comparison of DOC flux from DwH oil photo-dissolution to Mississippi River export

The total DOC produced from DwH surface slicks was compared to the average of the 2000's decadal averages of the Mississippi River DOC exports to the GoM during spring and summer (*69*). We chose to use the average of the spring and summer exports because the DwH spill resulted in surface slicks from April to August.

Appendix S7: DOC saturation test

We tested whether DOC production might be artificially lowered in our experimental system due to saturation of the aqueous solution in the water volumes we used, resulting in artificially low AQYs. For all wavelengths and all photon doses, our irradiated oil-to-water ratios were similar (2.4 ± 0.1 mg oil/mL water, n = 100). If the oil photo-dissolution products reach saturation at this oil-to-water ratio, then the DOC we measured in lab would be suppressed compared to the DOC that would be produced in a spill scenario in the ocean. For one wavelength (369 nm) we investigated DOC production as a function of oil-to-water ratio, measuring DOC ratios that were both lower (1 mg oil/mL water) and higher (4 mg oil/mL water) than that of our samples. If saturation occurred in this oil-to-water ratio range, we would expect DOC to remain constant as a function of oil-to-water ratio. Instead, we found that DOC decreased by nearly three-fold between a ratio of 1 mg/mL and 4 mg/mL (Fig. S4). Assuming that the oil photo-dissolution products at various wavelengths have similar solubilities, this result shows that DOC production was not limited by saturation in our experimental system.

Appendix S8: Biological contributions to DOC production

If microbes were present and active in our oil films, biodegradation could contribute to the DOC production we observed. To test if biodegradation could have contributed to DOC production in our experimental system, we analyzed two well-established indicators of biological oxidation, the heptadecane (n-C_{17}) to pristane ratio and the octadecane (n-C_{18}) to phytane ratio (*59*), in an oil sample allowed to weather in the dark for the longest experimental exposure period (7.5 days). We compared the ratios to those found in a sample which was stored capped in the dark (no weathering). The principle of these indicators is as follows: Straight-chain heptadecane and octadecane are more bioavailable than their branched isomers pristane and phytane (respectively). Thus, a decrease in the quantity of heptadecane relative to pristane or octadecane relative to phytane over the 7.5-day weathering period indicates biodegradation.

A comparison of the n-C_{17}/pristane and n-C_{18}/phytane ratios in the un-weathered and weathered dark control samples showed no evidence of biodegradation over the longest experimental exposure period of 7.5 days. The analyses of n-C_{17}/pristane and n-C_{18}/phytane were conducted by GC x GC-FID as discussed previously (*5*, *70*). Triplicate analyses resulted in ratios shown in Table S4. There was no significant difference in either ratio between the weathered and un-weathered treatments (two-sample t-test assuming unequal variance, $n = 3$, $p > 0.05$). Thus, no microbial activity was apparent in our dark control samples, suggesting that our experiments were not affected by biodegradation in any substantial manner. This quantitative, experimental evidence is consistent with several widely accepted controls of oil biodegradation that are relevant to our study design.

First, the most microbially-labile, short-chain hydrocarbons (< n-C_{16}) present in crude oil were removed prior to irradiation during the evaporative weathering step, in which we removed 35% of the oil by mass to simulate evaporation of volatile components in a spill scenario.

Second, even if there were labile compounds available for microbes (either native to the oil or produced photochemically), water and nutrients are required for microbial activity. Neither water nor nutrients were present in the study design. Macondo oil contains trace amounts of water (~0.02% by mass, see Ward et al., 2019 (*66*)), all glassware was combusted and/or cleaned with dichloromethane prior to use, and no water or nutrients were introduced during the irradiation. Previous work has found that, during DwH, nutrient limitation in the Gulf of Mexico prevented microbial growth on floating oil slicks over the timescale of weeks to months (*5*, *42, 57*). Collectively, it is reasonable to assume that water and nutrient limitation would also prevent microbial growth on oil films in the absence of water and nutrients in a laboratory setting over the timescale of 1-7.5 days.

Lastly, we expect that any microbial growth in the oil samples would be substantially inhibited by the high and continuous irradiance output by the LED light sources *(71).* Particularly in the UV region, the experimental light exposures resulted in irradiances that were > 1.5x higher than the irradiance delivered to the sea surface by a similar waveband of sunlight on a clear-skied summer day in the Gulf of Mexico, for 1-7.5 days of continuous laboratory exposure. At the shortest wavelength (278 nm), our irradiances were 14x higher than a similar waveband of natural sunlight. As high-intensity UV irradiation is used for microbial disinfection in water treatment (*72*), it’s unlikely that microbial growth would be favored under these conditions.

Furthermore, if microbial growth did substantially contribute to the formation of water-soluble products, we would expect to see lower production in the UV region compared to the visible region due to inhibition of microbial growth under the UV light. We observed the opposite of this expectation (Fig. 2), further suggesting that microbial activity was not a contributing factor to DOC production in our experiments.

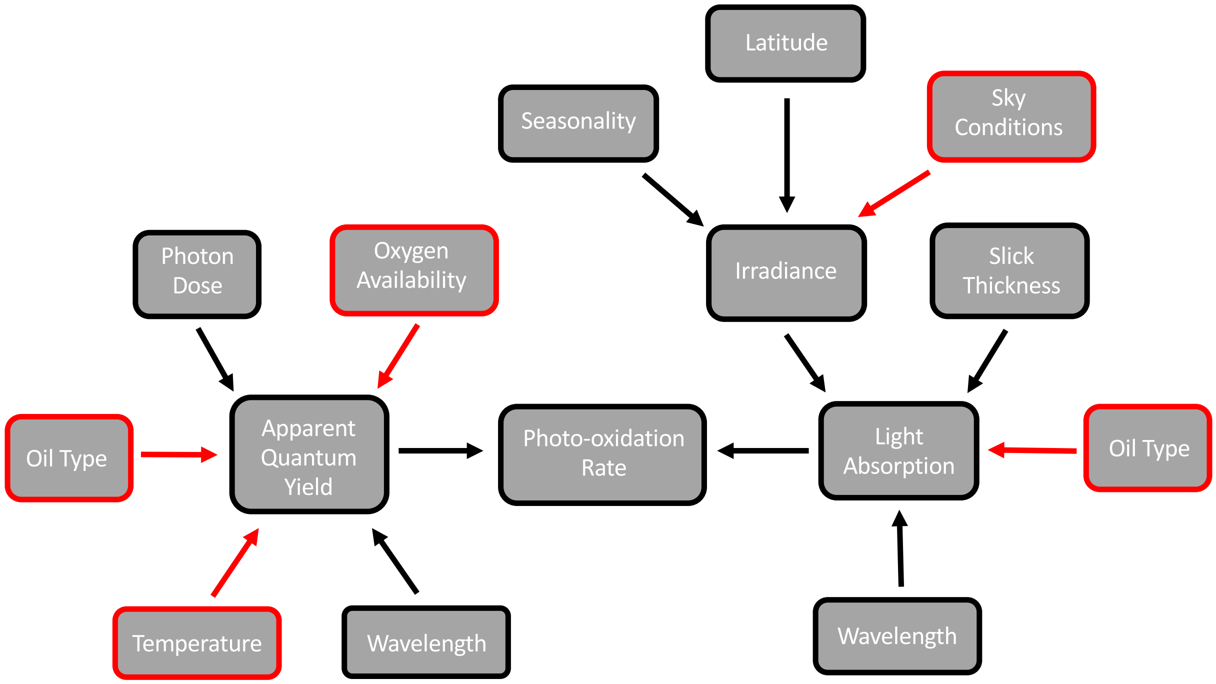

Figure S1. Variables impacting oil photo-oxidation rate. Variables outlined in black were investigated in this study, while variables outlined in red should be addressed in future research.

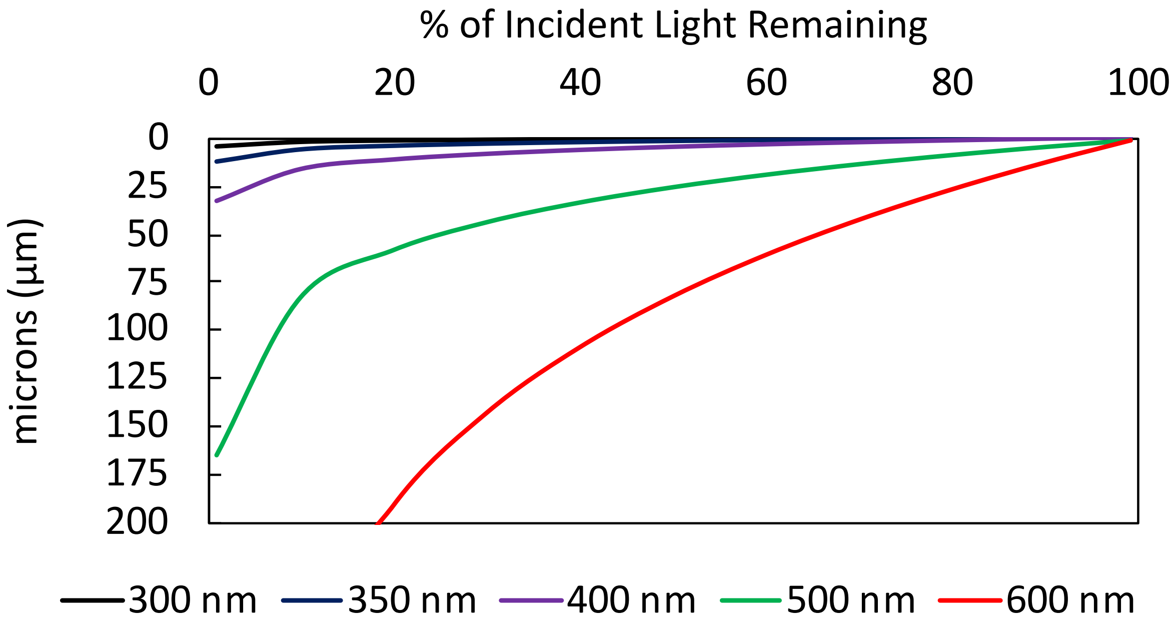

Figure S2. Percentage of light of various wavelengths remaining as a function of depth in an oil film.

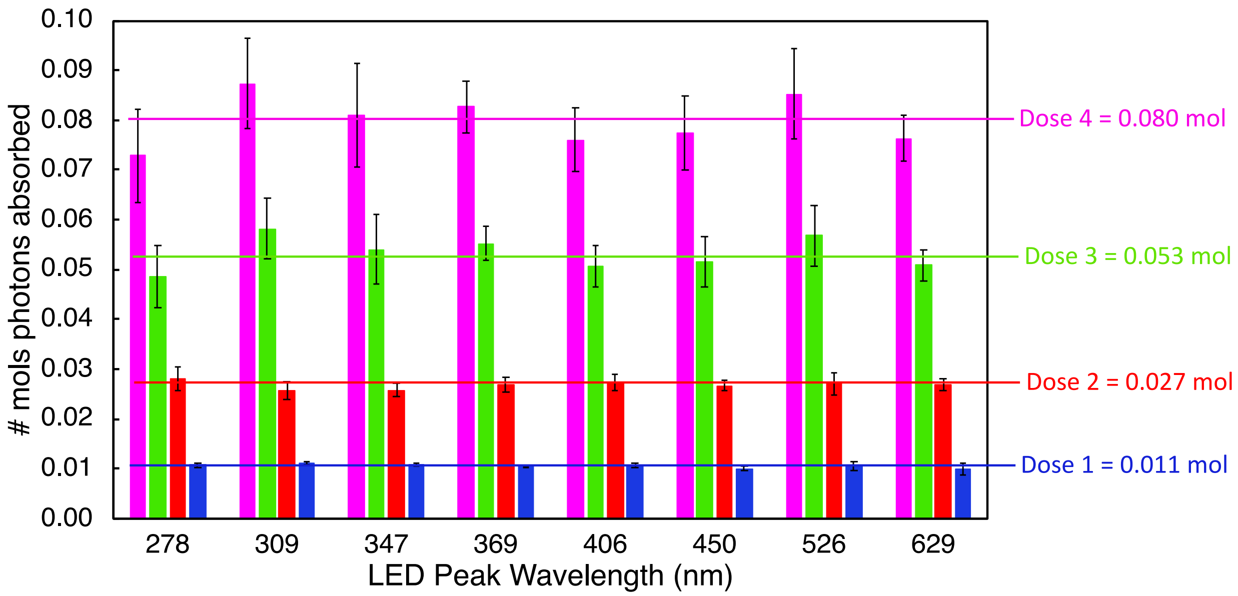

Figure S3. Mols of photons absorbed by oil under each LED for four experimental trials. In each trial, the LEDs were powered to achieve a target photon dose (horizontal lines) that was similar across all wavelengths. Error bars are standard errors (n = 5). Target photon doses for each trial were: Dose 1 = 0.011 mol photons, Dose 2 = 0.027 mol photons (2.5x Dose 1), Dose 3 = 0.053 mol photons (5x Dose 1), Dose 4 = 0.080 mol photons (7.5x Dose 1).

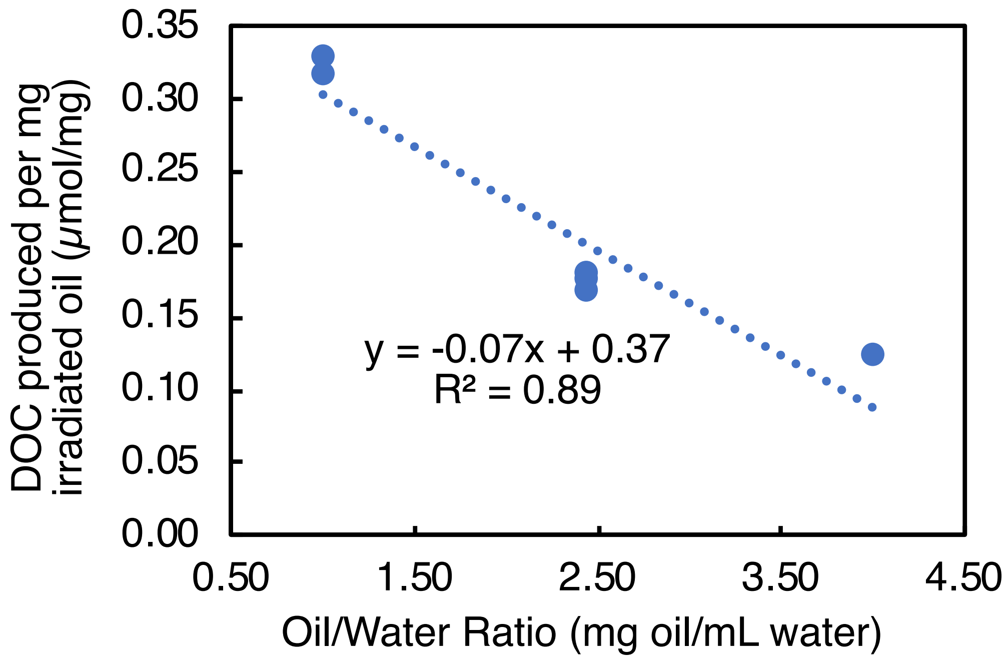

Figure S4. DOC produced due to irradiation by the 369 nm LED (Case 3 photon dose) as a function of oil-to-water ratio. The equation corresponds to a linear regression. The middle oil-to-water ratio of 2.5 was used to generate all AQY spectra.

Table S1. Tests included in sensitivity analysis and input variables

	Input Variable			
**Variable Tested**	**Φ_λ (mol DOC mol^{-1} photons)**	**$E_{o\lambda}$ (mol photons m^{-2} d^{-1})**	**a_λ (m^{-1})**	**z (μm)**
**Slick Thickness (z)**	Photon Dose 1 AQYs (Figure 1(A)) 280-700 nm	30N Jun 21 Daily Average Irradiance reference spectrum 280-700 nm*	Experimentally determined absorbance coefficients for Macondo oil 280-700 nm	0.1-1000
**Wavelength range (nm)**	Photon Dose 1 AQYs (Figure 1(A)) 280-400 nm OR 400-700 nm	30N Jun 21 Daily Average Irradiance reference spectrum 280-400 nm or 400-700 nm*	Experimentally determined absorbance coefficients for Macondo oil 280-400 nm or 400-700 nm	1
**Season**	Photon Dose 1 AQYs (Figure 1(A)) 280-700 nm	30N Mar 20, Jun 21, Sept 22, or Dec 22 Daily Average Irradiance reference spectra 280-700 nm*	Experimentally determined absorbance coefficients for Macondo oil 280-700 nm	1
**Latitude**	Photon Dose 1 AQYs (Figure 1(A)) 280-700 nm	0°N, 10°N, 20°N, 30°N, 40°N, 50°N, or 60°N Jun 21 Daily Average Irradiance reference spectrum 280-700 nm*	Experimentally determined absorbance coefficients for Macondo oil 280-700 nm	1
**Photon Dose**	Photon Dose 1, 2, 3, or 4 AQYs (Figure 1(A-D)) 280-700 nm	30N Jun 21 Daily Average Irradiance reference spectrum 280-700 nm*	Experimentally determined absorbance coefficients for Macondo oil 280-700 nm	1

*Solar irradiance reference spectra from Apell & McNeill *(32).*

Table S2. Solar exposure times (days) required for 20-nm wavebands of natural sunlight to achieve the photon doses investigated in this study for a 70-µm thick oil slick on the sea surface at 30°N.

Waveband (nm)	Dose 1	Dose 2	Dose 3	Dose 4	Environmental AQYs
280-299	7975	20344	40480	60721	7975
300-319	25	64	127	190	25
320-339	6	16	32	48	16
340-359	5	12	24	35	12
360-379	4	9	18	27	9
380-399	3	9	17	26	9
400-419	2	5	10	14	14
420-439	2	5	9	13	13
440-459	1	4	7	11	11
460-479	1	3	7	10	10
480-499	1	4	7	11	11
500-519	2	4	8	12	12
520-539	2	4	8	13	13
540-559	2	5	9	14	14
560-579	2	5	11	16	16
580-599	3	6	13	19	19
600-619	3	7	14	21	21
620-639	3	8	17	25	17
640-659	4	10	20	30	20
660-679	4	11	22	33	22
680-699	5	14	28	42	28

Table S3. Context to understand the presentation of fractions and fluxes in DwH mass balance studies used to produce Fig. 5

Study	Fate processes covered	Fate process presented as a fraction or mass flux?	Fraction of what?
(20)	Direct recovery, natural dispersion, evaporation combined with dissolution, chemical dispersion, burning, skimming, "other oil"	Both	Total amount of oil released before direct recovery (5 million barrels)
(45)	Evaporation	Both	Total cumulative surfacing hydrocarbons (oil + gas)
(44)	Stranding	Both	Total cumulative surface oil
(47)	Surfacing, evaporation,	Fraction	Total cumulative oil, specified as 564,717-1,014,964 metric tons, released (not specified if before or after direct recovery)
(21)	Stranding, sedimentation, biodegradation of soluble compounds, biodegradation of insoluble compounds, recovery/burning	Fraction	Total cumulative hydrocarbons (oil + gas) released before direct recovery (5 million barrels)
(46)	Dissolution in deep water, dissolution in upper water column, surfacing, evaporation, liquid petroleum in deep water	Fraction	Total cumulative hydrocarbons (oil + gas) released after direct recovery (4.2 million barrels)
(43)	MOSSFA	Mass flux	N/A
(48)	Surfacing, evaporation	Mass	Total cumulative hydrocarbons released after direct recovery (530,000 tons oil + 170,000 tons natural gas)
(22)	Surfacing, evaporation, entrainment, burning/skimming, MOSSFA, dissolution in deep water	Fraction	Total amount of oil released after direct recovery (4.2 million barrels)

Table S4. n-C_{17}/pristane and n-C_{18}/phytane ratios for 35% evaporated Macondo oil (T zero) and 30% evaporated Macondo oil weathered in the dark for 7.5 days (T 7.5), and statistics

	**n-C_{17}/pristane**		**n-C_{18}/phytane**	
**Replicate**	**T zero**	**T 7.5**	**T zero**	**T 7.5**
1	1.694	1.707	2.453	2.481
2	1.701	1.697	2.449	2.508
3	1.681	1.678	2.468	2.482
Average	1.692	1.694	2.457	2.490
Standard Deviation	0.010	0.015	0.010	0.016
p-value (two-tail t-test assuming unequal variance)	0.8540		0.0509	
% difference between means	0.1		1.4	

REFERENCES AND NOTES

1. J. R. Payne, C. R. Phillips, Photochemistry of petroleum in water. *Environ. Sci. Technol.* **19**, 569–579 (1985).

2. C. P. Ward, E. B. Overton, How the 2010 Deepwater Horizon spill reshaped our understanding of crude oil photochemical weathering at sea: A past, present, and future perspective. *Environ. Sci.* **22**, 1125–1138 (2020).

3. D. E. Nicodem, C. L. B. Guedes, M. Conceição, Z. Fernandes, D. Severino, R. J. Correa, M. C. Coutinho, J. Silva, Photochemistry of petroleum. *Prog. React. Kinet. Mech.* **26**, 219–238 (2001).

4. E. B. Overton, J. L. Laseter, S. W. Mascarella, C. Raschke, I. Nuiry, J. W. Farrington, Photo-chemical oxidation of Ixtoc-I oil. In: *Proceedings of a Symposium on Preliminary Results from the September 1979 Researcher/Pierce IXTOC I Cruise. Key Biscayne, Florida, June 9–10, 1980* (Office of Marine Pollution Assessment, National Oceanic and Atmospheric Administration, U.S. Dept. of Commerce,1980), pp. 341–383.

5. C. P. Ward, C. M. Sharpless, D. L. Valentine, D. P. French-McCay, C. Aeppli, H. K. White, R. P. Rodgers, K. M. Gosselin, R. K. Nelson, C. M. Reddy, Partial photochemical oxidation was a dominant fate of deepwater horizon surface oil. *Environ. Sci. Technol.* **52**, 1797–1805 (2018).

6. C. P. Ward, C. J. Armstrong, R. N. Conmy, D. P. French-McCay, C. M. Reddy, Photochemical oxidation of oil reduced the effectiveness of aerial dispersants applied in response to the deepwater horizon spill. *Environ. Sci. Technol. Lett.* **5**, 226–231 (2018).

7. C. Aeppli, R. F. Swarthout, G. W. O'Neil, S. D. Katz, D. Nabi, C. P. Ward, R. K. Nelson, C. M. Sharpless, C. M. Reddy, How persistent and bioavailable are oxygenated deepwater horizon oil transformation products? *Environ. Sci. Technol.* **52**, 7250–7258 (2018).

8. B. M. Ruddy, M. Huettel, J. E. Kostka, V. V. Lobodin, B. J. Bythell, A. M. McKenna, C. Aeppli, C. M. Reddy, R. K. Nelson, A. G. Marshall, R. P. Rodgers, Targeted petroleomics: Analytical investigation of macondo well oil oxidation products from pensacola beach. *Energy Fuel* **28**, 4043–4050 (2014).

9. H. K. White, R. N. Conmy, I. R. MacDonald, C. M. Reddy, Methods of oil detection in response to the Deepwater Horizon oil spill. *Oceanography* **29**, 76–87 (2016).

10. P. Zito, D. C. Podgorski, T. Bartges, F. Guillemette, J. A. Roebuck, R. G. M. Spencer, R. P. Rodgers, M. A. Tarr, Sunlight-induced molecular progression of oil into oxidized oil soluble species, interfacial material, and dissolved organic matter. *Energy Fuel* **34**, 4721–4726 (2020).

11. M. Freegarde, C. G. Hatchard, C. A. Parker, Oil spilt at sea: Its identification, determination, and ultimate fate. *Lab. Practice* **20**, 35–40 (1971).

12. R. Burwood, G. C. Speers, Photo-oxidation as a factor in the environmental dispersal of crude oil. *Estuar. Coast. Mar. Sci.* **2**, 117–135 (1974).

13. H. P. Hansen, Photochemical degradation of petroleum hydrocarbon surface films on seawater. *Mar. Chem.* **3**, 183–195 (1975).

14. M. L. Spaulding, A state-of-the-art review of oil spill trajectory and fate modeling. *Oil Chem. Pollut.* **4**, 39–55 (1988).

15. M. Reed, O. Johansen, P. Brandvik, P. Daling, A. Lewis, R. Fiocco, D. Mackay, R. Prentki, Oil spill modeling towards the close of the 20th century: Overview of the state of the art. *Spill Sci. Technol. Bull.* **5**, 3–16 (1999).

16. D. P. French-McCay, Oil spill impact modeling: Development & validation. *Environ. Toxicol. Chem.* **23**, 2441–2456 (2004).

17. D. A. Wolfe, M. J. Hameedi, J. A. Galt, G. Watabayashi, J. Short, C. O'Claire, S. Rice, J. Michel, J. R. Payne, J. Braddock, S. Hanna, D. Sale, The fate of the oil spilled from the Exxon Valdez. *Environ. Sci. Technol.* **28**, 561–568 (1994).

18. G. S. Douglas, E. H. Owens, J. Hardenstine, R. C. Prince, The OSSA II pipeline oil spill: The character and weathering of the spilled oil. *Spill Sci. Technol. Bull.* **7**, 135–148 (2002).

19. Transportation Research Board and National Research Council, *Oil in the Sea III: Inputs, Fates, and Effects* (The National Academies Press, 2003); www.nap.edu/catalog/10388/oil-in-the-sea-iii-inputs-fates-and-effects.

20. B. Lehr, S. Bristol, A. Possolo, "Oil budget calculator: Deepwater Horizon" (Report to the National Incident Command: Federal Interagency Solutions Group, 2010).

21. U. Passow, R. Hetland, What happened to all of the oil? *Oceanography* **29**, 88–95 (2016).

22. D. P. French-McCay, K. Jayko, Z. Li, M. L. Spaulding, D. Crowley, D. Mendelsohn, M. Horn, T. Isaji, Y. H. Kim, J. Fontenault, J. J. Rowe, Oil fate and mass balance for the Deepwater Horizon oil spill. *Mar. Pollut. Bull.* **171**, 112681 (2021).

23. O. C. Mullins, E. Y. Sheu, *Structures and Dynamics of Asphaltenes* (Springer Science + Business Media, 1998).

24. C. P. Ward, J. C. Bowen, D. H. Freeman, C. M. Sharpless, Rapid and reproducible characterization of the wavelength dependence of aquatic photochemical reactions using light-emitting diodes. *Environ. Sci. Technol. Lett.* **8**, 437–442 (2021).

25. J. C. Bowen, C. P. Ward, G. W. Kling, R. M. Cory, Arctic amplification of global warming strengthened by sunlight oxidation of permafrost Carbon to CO 2. *Geophys. Res. Lett.* **47**, e87085 (2020).

26. D. J. Kieber, G. W. Miller, P. J. Neale, K. Mopper, Wavelength and temperature-dependent apparent quantum yields for photochemical formation of hydrogen peroxide in seawater. *Environ. Sci. Process. Impacts* **16**, 777–791 (2014).

27. R. M. Garrett, I. J. Pickering, C. E. Haith, R. C. Prince, Photooxidation of crude oils. *Environ. Sci. Technol.* **32**, 3719–3723 (1998).

28. B. E. Finch, E. S. Stefansson, C. J. Langdon, S. M. Pargee, S. M. Blunt, S. J. Gage, W. A. Stubblefield, Photo-enhanced toxicity of two weathered Macondo crude oils to early life stages of the eastern oyster (Crassostrea virginica). *Mar. Pollut. Bull.* **113**, 316–323 (2016).

29. W. L. Miller, R. G. Zepp, Photochemical production of dissolved inorganic carbon from terrestrial organic matter: Significance to the oceanic organic carbon cycle. *Geophys. Res. Lett.* **22**, 417–420 (1995).

30. R. M. Cory, C. P. Ward, B. C. Crump, G. W. Kling, Sunlight controls water column processing of carbon in arctic fresh waters. *Science* **345**, 925–928 (2014).

31. BP Gulf Science Data, “Chemical analysis and physical properties of weathered, unweathered, and surrogate crude oils from the Deepwater Horizon accident in the Gulf of Mexico, July 2010 to January 2011” (2016); doi:10.7266/N7R78CM9.

32. J. N. Apell, K. McNeill, Updated and validated solar irradiance reference spectra for estimating environmental photodegradation rates. *Environ. Sci. Process. Impacts* **21**, 427–437 (2019).

33. I. R. MacDonald, O. Garcia-Pineda, A. Beet, S. Daneshgar Asl, L. Feng, G. Graettinger, D. French-McCay, J. Holmes, C. Hu, F. Huffer, I. Leifer, F. Muller-Karger, A. Solow, M. Silva, G. Swayze, Natural and unnatural oil slicks in the Gulf of Mexico. *J. Geophys. Res.* **120**, 8364–8380 (2015).

34. H. Gao, R. G. Zepp, Factors influencing photoreactions of dissolved organic matter in a coastal river of the Southeastern United States. *Environ. Sci. Technol.* **32**, 2940–2946 (1998).

35. S. S. Andrews, S. Caron, O. C. Zafiriou, Photochemical oxygen consumption in marine waters: A major sink for colored dissolved organic matter? *Limnol. Oceanogr.* **45**, 267–277 (2000).

36. H. E. Reader, W. L. Miller, The efficiency and spectral photon dose dependence of photochemically induced changes to the bioavailability of dissolved organic carbon. *Limnol. Oceanogr.* **59**, 182–194 (2014).

37. L. C. Powers, W. L. Miller, Photochemical production of CO and CO2 in the Northern Gulf of Mexico: Estimates and challenges for quantifying the impact of photochemistry on carbon cycles. *Mar. Chem.* **171**, 21–35 (2015).

38. O. Garcia-Pineda, G. Staples, C. E. Jones, C. Hu, B. Holt, V. Kourafalou, G. Graettinger, L. DiPinto, E. Ramirez, D. Streett, J. Cho, G. A. Swayze, S. Sun, D. Garcia, F. Haces-Garcia, Classification of oil spill by thicknesses using multiple remote sensors. *Remote Sens. Environ.* **236**, 111421 (2020).

39. D. E. Nicodem, M. C. Z. Fernandes, C. L. B. Guedes, J. Correa, Photochemical processes and the environmental impact of petroleum spills. *Biogeochemistry* **39**, 121–138 (1997).

40. L. C. Smith, S. R. Stephenson, New Trans-Arctic shipping routes navigable by midcentury. *Proc. Natl. Acad. Sci. U.S.A.* **110**, E1191–E1195 (2013).

41. S. R. Stephenson, L. W. Brigham, L. C. Smith, Marine accessibility along Russia’s Northern Sea Route. *Polar Geogr.* **37**, 111–133 (2014).

42. S. A. Stout, J. R. Payne, S. D. Emsbo-Mattingly, G. Baker, Weathering of field-collected floating and stranded Macondo oils during and shortly after the Deepwater Horizon oil spill. *Mar. Pollut. Bull.* **105**, 7–22 (2016).

43. S. A. Stout, C. R. German, Characterization and flux of marine oil snow settling toward the seafloor in the northern Gulf of Mexico during the Deepwater Horizon incident: Evidence for input from surface oil and impact on shallow shelf sediments. *Mar. Pollut. Bull.* **129**, 695–713 (2018).

44. M. C. Boufadel, A. Abdollahi-Nasab, X. Geng, J. Galt, J. Torlapati, Simulation of the landfall of the deepwater horizon oil on the shorelines of the gulf of Mexico. *Environ. Sci. Technol.* **48**, 9496–9505 (2014).

45. T. B. Ryerson, R. Camilli, J. D. Kessler, E. B. Kujawinski, C. M. Reddy, D. L. Valentine, E. Atlas, D. R. Blake, J. de Gouw, S. Meinardi, D. D. Parrish, J. Peischl, J. S. Seewald, C. Warneke, Chemical data quantify Deepwater Horizon hydrocarbon flow rate and environmental distribution. *Proc. Natl. Acad. Sci. U.S.A.* **109**, 20246–20253 (2012).

46. J. Gros, S. A. Socolofsky, A. L. Dissanayake, I. Jun, L. Zhao, M. C. Boufadel, C. M. Reddy, J. S. Arey, Petroleum dynamics in the sea and influence of subsea dispersant injection during Deepwater Horizon. *Proc. Natl. Acad. Sci. U.S.A.* **114**, 10065–10070 (2017).

7. S. B. Joye, A. Bracco, T. M. Özgökmen, J. P. Chanton, M. Grosell, I. R. MacDonald, E. E. Cordes, J. P. Montoya, U. Passow, The Gulf of Mexico ecosystem, six years after the Macondo oil well blowout. *Deep-Sea Res. II Top. Stud. Oceanogr.* **129**, 4–19 (2016).

48. E. B. Kujawinski, C. M. Reddy, R. P. Rodgers, J. C. Thrash, D. L. Valentine, H. K. White, The first decade of scientific insights from the Deepwater Horizon oil release. *Nat. Rev. Earth Environ.* **1**, 237–250 (2020).

49. E. J. D'Sa, E. B. Overton, S. E. Lohrenz, K. Maiti, R. E. Turner, A. Freeman, Changing dynamics of dissolved organic matter fluorescence in the Northern gulf of Mexico following the Deepwater Horizon oil spill. *Environ. Sci. Technol.* **50**, 4940–4950 (2016).

50. H. P. Bacosa, D. L. Erdner, Z. Liu, Differentiating the roles of photooxidation and biodegradation in the weathering of Light Louisiana Sweet crude oil in surface water from the Deepwater Horizon site. *Mar. Pollut. Bull.* **95**, 265–272 (2015).

51. R. C. Prince, J. D. Butler, A. D. Redman, The rate of crude oil biodegradation in the sea. *Environ. Sci. Technol.* **51**, 1278–1284 (2017).

52. T. K. Dutta, S. Harayama, Fate of crude oil by the combination of photooxidation and biodegradation. *Environ. Sci. Technol.* **34**, 1500–1505 (2000).

53. B. H. Harriman, P. Zito, D. C. Podgorski, M. A. Tarr, J. M. Suflita, Impact of photooxidation and biodegradation on the fate of oil spilled during the deepwater Horizon incident: Advanced stages of weathering. *Environ. Sci. Technol.* **51**, 7412–7421 (2017).

54. R. A. Larson, L. L. Hunt, D. W. Blankenship, Formation of toxic products from a #2 fuel oil by photooxidation. *Environ. Sci. Technol.* **11**, 492–496 (1977).

55. R. A. Larson, T. L. Bott, L. L. Hunt, K. Rogenmuser, Photooxidation products of a fuel oil and their antimicrobial activity. *Environ. Sci. Technol.* **13**, 965–969 (1979).

56. P. Zito, D. C. Podgorski, J. Johnson, H. Chen, R. P. Rodgers, F. Guillemette, A. M. Kellerman, R. G. M. Spencer, M. A. Tarr, Molecular-level composition and acute toxicity of photosolubilized petrogenic carbon. *Environ. Sci. Technol.* **53**, 8235–8243 (2019).

57. B. R. Edwards, C. M. Reddy, R. Camilli, C. A. Carmichael, K. Longnecker, B. A. S. Van Mooy, Rapid microbial respiration of oil from the *Deepwater Horizon* spill in offshore surface waters of the Gulf of Mexico. *Environ. Res. Lett.* **6**, 035301 (2011).

58. C. P. Ward, C. J. Armstrong, A. N. Walsh, J. H. Jackson, C. M. Reddy, Sunlight converts polystyrene to carbon dioxide and dissolved organic carbon. *Environ. Sci. Technol. Lett.* **6**, 669–674 (2019).

59. J. Gros, C. M. Reddy, C. Aeppli, R. K. Nelson, C. A. Carmichael, J. S. Arey, Resolving biodegradation patterns of persistent saturated hydrocarbons in weathered oil samples from the deepwater horizon disaster. *Environ. Sci. Technol.* **48**, 1628–1637 (2014).

60. C. M. Reddy, J. S. Arey, J. S. Seewald, S. P. Sylva, K. L. Lemkau, R. K. Nelson, C. A. Carmichael, C. P. McIntyre, J. Fenwick, G. T. Ventura, B. A. S. Van Mooy, R. Camilli, Composition and fate of gas and oil released to the water column during the Deepwater Horizon oil spill. *Proc. Natl. Acad. Sci. U.S.A.* **109**, 20229–20234 (2012).

61. B. I. Barton, Y.-D. Lenn, C. Lique, Observed atlantification of the barents sea causes the polar front to limit the expansion of winter sea ice. *J. Phys. Oceanogr.* **48**, 1849–1866 (2018).

62. J. Hong, H. Xie, L. Guo, G. Song, Carbon monoxide photoproduction: Implications for photoreactivity of arctic permafrost-derived soil dissolved organic matter. *Environ. Sci. Technol.* **48**, 9113–9121 (2014).

63. K. N. Bridges, C. R. Lay, M. M. Alloy, M. L. Gielazyn, J. M. Morris, H. P. Forth, R. Takeshita, C. L. Travers, J. T. Oris, A. P. Roberts, Estimating incident ultraviolet radiation exposure in the northern Gulf of Mexico during the Deepwater Horizon oil spill. *Environ. Toxicol. Chem.* **37**, 1679–1687 (2018).

64. M. Fingas, in *Oil Spill Science and Technology* (Elsevier, 2011), pp. 51–59.

65. F. Thominette, J. Verdu, Photo-oxidative behaviour of crude oils relative to sea pollution. *Mar. Chem.* **15**, 91–104 (1984).

66. C. P. Ward, C. M. Sharpless, D. L. Valentine, C. Aeppli, K. M. Sutherland, S. D. Wankel, C. M. Reddy, Oxygen isotopes (δ18O) trace photochemical hydrocarbon oxidation at the sea surface. *Geophys. Res. Lett.* **46**, 6745–6754 (2019).

67. M. K. McNutt, R. Camilli, T. J. Crone, G. D. Guthrie, P. A. Hsieh, T. B. Ryerson, O. Savas, F. Shaffer, Review of flow rate estimates of the Deepwater Horizon oil spill. *Proc. Natl. Acad. Sci. U.S.A.* **109**, 20260–20267 (2012).

68. S. C. Johannessen, W. L. Miller, J. J. Cullen, Calculation of UV attenuation and colored dissolved organic matter absorption spectra from measurements of ocean color. *J. Geophys. Res.* **108**, 3301 (2003).

69. W. Ren, H. Tian, W.-J. Cai, S. E. Lohrenz, C. S. Hopkinson, W.-J. Huang, J. Yang, B. Tao, S. Pan, R. He, Century-long increasing trend and variability of dissolved organic carbon export from the Mississippi River basin driven by natural and anthropogenic forcing. *Global Biogeochem. Cycles* **30**, 1288–1299 (2016).

70. R. K. Nelson, C. Aeppli, J. Samuel, H. Chen, R. B. Gaines, K. Grice, J. Gros, G. J. Hall, H. H. F. Koolen, K. L. Lemkau, A. M. McKenna, C. M. Reddy, R. P. Rodgers, R. F. Swarthout, D. L. Valentine, H. K. White, *Standard Handbook Oil Spill Environental Forensics* (Elsevier, 2016).

71. C. P. Ward, S. G. Nalven, B. C. Crump, G. W. Kling, R. M. Cory, Photochemical alteration of organic carbon draining permafrost soils shifts microbial metabolic pathways and stimulates respiration. *Nat. Commun.* **8**, 772 (2017).

72. K. Song, M. Mohseni, F. Taghipour, Application of ultraviolet light-emitting diodes (UV-LEDs) for water disinfection: A review. *Water Res.* **94**, 341–349 (2016).
